# Solubility-mediated sustained release enabling nitrate additive in carbonate electrolytes for stable lithium metal anode

**DOI:** 10.1038/s41467-018-06077-5

**Published:** 2018-09-07

**Authors:** Yayuan Liu, Dingchang Lin, Yuzhang Li, Guangxu Chen, Allen Pei, Oliver Nix, Yanbin Li, Yi Cui

**Affiliations:** 1 Department of Materials Science and Engineering, Stanford University, Stanford, CA 94305 USA; 2 Stanford Institute for Materials and Energy Sciences, SLAC National Accelerator Laboratory, 2575 Sand Hill Road, Menlo Park, CA 94025 USA

## Abstract

The physiochemical properties of the solid-electrolyte interphase, primarily governed by electrolyte composition, have a profound impact on the electrochemical cycling of metallic lithium. Herein, we discover that the effect of nitrate anions on regulating lithium deposition previously known in ether-based electrolytes can be extended to carbonate-based systems, which dramatically alters the nuclei from dendritic to spherical, albeit extremely limited solubility. This is attributed to the preferential reduction of nitrate during solid-electrolyte interphase formation, and the mechanisms behind which are investigated based on the structure, ion-transport properties, and charge transfer kinetics of the modified interfacial environment. To overcome the solubility barrier, a solubility-mediated sustained-release methodology is introduced, in which nitrate nanoparticles are encapsulated in porous polymer gel and can be steadily dissolved during battery operation to maintain a high concentration at the electroplating front. As such, effective dendrite suppression and remarkably enhanced cycling stability are achieved in corrosive carbonate electrolytes.

## Abstract (web summary)

The solid-electrolyte interphase (SEI) is one of the governing factors for the reversibility of Li metal anode. Here, the authors reveal the impact of nitrate additive on the SEI in carbonate electrolytes, and demonstrate a method to overcome the solubility limitation of nitrate.

## Introduction

The morphology of electrodeposited metals is dictated to a great extent by the physiochemical characteristics at the metal/electrolyte interface, including ion transport, interfacial energy, and mechanics, etc.^1^. This especially stands for the electrochemical plating of metallic lithium (Li), the ultimate anode for next-generation batteries with highest specific capacity (3860 mAh g^–1^) and lowest electrode potential of all possible alternatives^2,3^. In particular, the electrodeposition of Li is complicated by the instantaneous formation of a resistive interfacial passivation (i.e. the solid-electrolyte interphase, SEI), originated from the parasitic reduction of electrolyte components by the highly reactive Li^4^. The chemical heterogeneity and mechanical instability of the SEI layer is generally believed to induce nonuniform ion flux, resulting in the formation of Li dendrites that could lead to internal short circuit and compromise battery safety, while the repeated breakdown and repair of SEI brings about continuous loss of active materials, giving rise to limited battery cycle life^5^.

Extensive research has been devoted to regulate the surface reactivity of Li metal. Among all the tactics explored to date (protective coatings^6,7^, nanostructured electrodes^8,9^, high-modulus separators^10,11^, etc.), tailoring the electrolyte composition is among the most essential and prominent paradigms, for it can directly impact the physiochemistries of the SEI layer, modifying the interfacial environment to alter Li deposition behavior^12–16^.

It is known that the choice of electrolyte solvents can already offer pronounced effect on Li deposition uniformity. In general, ether-based electrolytes demonstrate relatively controlled deposition and thus high Coulombic efficiency (CE), ascribed to the formation of oligomeric SEI with superior flexibility^17^. The application of ether electrolytes, however, is severely hampered by its high flammability and low oxidation potential (anodic decomposition at <4 V vs Li^+^/Li)^18^, mismatched with the safety benchmark and the emerging high-voltage cathodes^19^. On the other hand, carbonate-based electrolytes exhibit lower flammability and higher anodic potential, and, therefore, are used exclusively in almost all the commercial Li-ion batteries. Nevertheless, they are highly corrosive to metallic Li, rendering aggravated dendrite formation and poor cycling efficiency. Fluorinated molecules, such as fluoroethylene carbonate (FEC), are frequently adopted as film-forming additives in carbonates, which is believed to promote the formation of LiF on Li surface as a favorable SEI passivation component^20–22^. Even with these additives, the Li anode CE remains deficient for practical applications, and the adverse effects of FEC decomposition at elevated temperatures bring concerns when being used in full-cells^23^. High salt concentration has recently arisen as another exemplary method to stabilize Li metal in carbonate electrolytes, the effect of which is originated from the unique solvation structure that reduces the solvent reactivity^24–26^. Nevertheless, its economic effectiveness, rheological properties, and ion transport need to be evaluated for practical applications. Thus, it is apparent that the development of new electrolyte reformulation strategies, which could circumvent the potential drawbacks of the existing ones, remains highly desirable.

Theoretically, if an additive is much more liable towards reduction than other electrolyte components to form an interfacial environment favorable for Li deposition, good electrochemical performance can be achieved regardless of the electrolyte systems (“electrolyte-agnostic” additive). Herein, we report the discovery of the pronounced effect of nitrate anions (NO_3_^–^) on the morphology and reversibility of Li deposition in carbonate-based electrolytes. Although nitrate additive is not unfamiliar to the community, as LiNO_3_ is regularly used in ethers for Li-sulfur batteries^27,28^, it remains being unexplored in carbonates partially due to its extremely low solubility. However, we observe that the preferential reduction of NO_3_^–^ during SEI formation in carbonates, albeit at very low concentration, could substantially alter the interfacial chemistry, resulting in spherical Li nuclei (instead of dendritic) and greatly improved CE exceeding the values in ethers. To further overcome the solubility limitation, a solubility-mediated sustained-release concept is proposed, where LiNO_3_ nanoparticles are dispersed in porous polymer backbone on anode surface and can be steadily dissolved during battery operation when soluble LiNO_3_ is consumed. With the maintained concentration of NO_3_^–^ on Li surface frontier, the anode CE can exceed 98% for over 200 cycles, and the cycle life of full-cells paired with LiNi_1/3_Mn_1/3_Co_1/3_O_2_ (NMC) is more than quadrupled, which is appreciable in corrosive carbonate electrolytes. Note that during the peer-review process of our manuscript, we noticed another work utilizing a slightly similar engineering method to overcome the solubility barrier of LiNO_3_ in carbonate electrolyte, which also resulted in improved Li anode CE^29^. Nevertheless, we are confident that our comprehensive work brings a significant amount of new knowledge complementary to the other study. Particularly, the mechanism of NO_3_^–^ in modifying the SEI properties is studied deliberately on the basis of cryo-electron microscopy (cryo-EM)^30,31^, ultramicroelectrode, X-ray photoelectron spectroscopy (XPS), and electrochemical impedance spectroscopy (EIS). The characterizations unravel a distinct structural change of the SEI from amorphous to bilayered configuration, a strong presence of nitrogen-containing species inside the SEI, and a greatly increased exchange current density of the Li^+^/Li couple with the addition of NO_3_^–^, and the effects of which are discussed in detail. This work not only enables uniform Li deposition with high reversibility in typical carbonate-based electrolytes without compromising the stability, ionic conductivity, viscosity, and cost of the electrolytes, but also provides fruitful insights on the exact role of nitrate additive at a fundamental level.

## Results

### Effects of nitrate on Li morphology in carbonate electrolyte

**Fig. 1:**
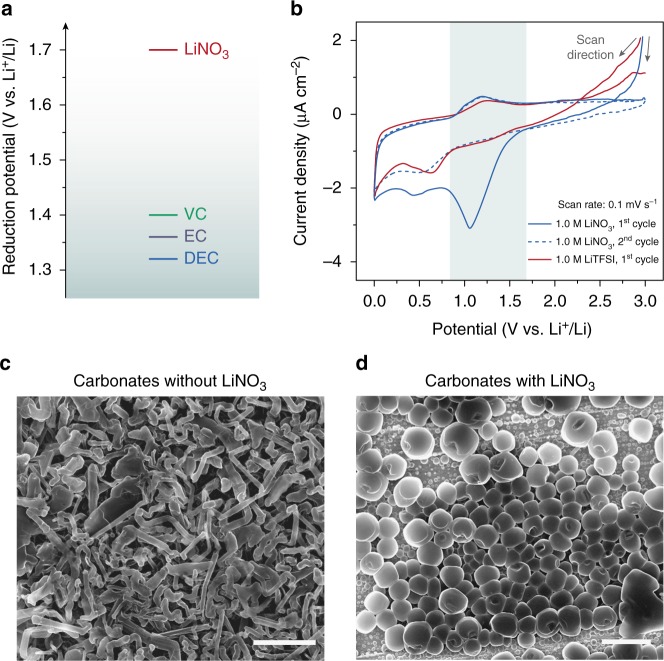
Preferential reduction of nitrate in carbonate electrolytes alters the Li nucleation morphology. **a** Experimental reduction potential of various electrolyte components (common solvents like EC and DEC; typical additive VC) on inert electrode reported in the literature^31^. **b** CV of DME-based electrolytes with 1.0 M LiNO_3_ or 1.0 M LiTFSI at a scan rate of 0.1 mV s^–1^. Stainless-steel was used as the working electrode and Li foil was used as the counter/reference electrode. SEM images of the Li nucleation morphology in 1.0 M LiPF_6_ EC/DEC electrolyte **c** without and **d** with saturated LiNO_3_. Scale bars, 2 μm. The deposition was carried out at a current density of 1 mA cm^–2^ and a capacity of 0.1 mAh cm^–2^

As frequently mentioned in the literature, the cathodic decomposition of NO_3_^–^ can start as early as ~1.7 V vs Li^+^/Li^32^, which is much higher than other carbonate-based electrolyte components, making it a promising candidate as “electrolyte-agnostic” additive (Fig. 1a)^33^. Therefore, if the preferential reduction of NO_3_^–^ can yield an SEI conducive to controlled Li deposition, the presence of NO_3_^–^ in carbonate electrolytes is highly likely to afford improved Li metal performance. To determine the exact reduction potential of NO_3_^–^, cyclic voltammogram (CV) of 1.0 M LiNO_3_ was obtained on a stainless-steel electrode at a scanning rate of 0.1 mV s^–1^. Dimethoxyethane (DME) was selected as the solvent for the CV scan, due to its stability against reduction (–1.68 V vs Li^+^/Li) and high LiNO_3_ solubility^34^. As can be observed in Fig. 1b, there is a deep cathodic current peak beginning at ~1.69 V vs Li/Li^+^ in the first CV cycle, which disappeared upon the second scan. And no similar reduction peak can be observed in the first CV cycle when LiNO_3_ was replaced by lithium bis(trifluoromethanesulfonyl)imide (LiTFSI). Thus, the irreversible cathodic current starting at ~1.69 V vs Li/Li^+^ can be assigned with confidence to the reduction of NO_3_^–^. The easy reduction of NO_3_^–^ participates as the frontier reaction during SEI formation and plays a decisive role in the physiochemical properties of the SEI. For this reason, the addition of LiNO_3_ in ether electrolytes can drastically change the Li deposition morphology from irregular to densely packed spheres, together with much improved CE (Supplementary Fig. 1).

To explore the effect of NO_3_^–^ in carbonate-based electrolytes, Li deposition on copper (Cu) foil was carried out using conventional 1.0 M lithium hexafluorophosphate (LiPF_6_) in 1:1 v/v ethylene carbonate (EC)/diethyl carbonate (DEC) electrolyte saturated with LiNO_3_. To our great surprise, although dendritic Li was observed in neat carbonate electrolyte at as early as the nucleation stage (0.1 mAh cm^–2^; Fig. 1c), uniform Li particles can be obtained in the presence of LiNO_3_ (Fig. 1d). Such impact on morphology is far more appreciable than conventional film-forming additives such as FEC and vinylene carbonate (VC), with which the Li deposition remained wire-shaped (Supplementary Fig. 2). Noticeably, the effect is indifference to the selection of cations and can be extended to other molecular species with N–O bond (e.g. tetradodecylammonium nitrate and sodium nitrite; Supplementary Fig. 3). The additive is also generally applicable to different carbonate systems (e.g. LiTFSI in propylene carbonate, PC, Supplementary Fig. 4).

In spite of the pronounced effect of NO_3_^–^ on Li nucleation morphology, the practicality of the additive is severely limited by its extremely low solubility in carbonates. Since NO_3_^–^ needs to be continuously consumed during SEI formation, its control over Li plating wears off quickly with increased deposition capacity (Supplementary Fig. 4).

### Overcoming the solubility barrier via sustained release

**Fig. 2:**
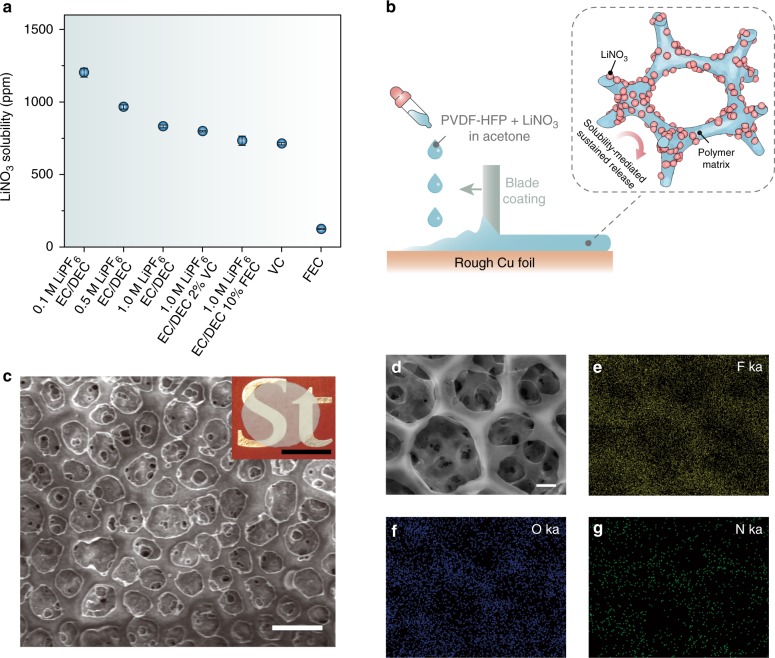
Fabrication of the solubility-mediated sustained-release film. **a** Solubility of LiNO_3_ in EC/DEC-based electrolytes with different LiPF_6_ concentrations and additives, in pure VC and in pure FEC. **b** Schematic illustrating the fabrication and working principle of LNO-SRF. **c** Top-view SEM image and photo image (inset) of LNO-SRF. Scale bar, 5 μm and inset scale bar, 1 cm. **d** High-magnification SEM image and **e**–**g** the corresponding EDX elemental mappings of LNO-SRF. Scale bar, 2 μm

The maximum concentration of NO_3_^–^ in various carbonate-based electrolytes was determined via colorimetry by cadmium reduction using a discrete analyzer (Fig. 2a). The solubility of LiNO_3_ in EC/DEC-based electrolytes is ~800 ppm (equivalent to ~0.012 M), which is at least one order of magnitude smaller than the additive concentration in ether electrolyte^27^. Reducing the LiPF_6_ concentration would increase the NO_3_^–^ solubility, ascribed to the “common-ion effect”. Though the dielectric constants of cyclic carbonate solvents are much higher than ethers, which intuitively shall result in better salt-dissolving capability, salt dissociation is also highly dependent upon the Gutmann donor number (DN) of the solvent, a parameter measuring the Lewis basicity^35^. In brief, for salt dissociation to occur, the electron donation from the solvent molecule to the cation has to exceed the electronic interaction between the cation and anion itself, which otherwise leads to strong contact ions pairs (Supplementary Fig. 5). However, the DN of cyclic carbonates (~15) is much lower than that of NO_3_^–^ (~22) and DME (~20)^36,37^, accounting for the low solubility of nitrate salts in carbonates.

To overcome the solubility limitation and render nitrate additive applicable in carbonates, we propose the strategy of solubility-mediated sustained release (Fig. 2b). Specifically, a freestanding membrane consists of LiNO_3_ dissolved in a polymeric matrix (termed LiNO_3_ sustained-release film, LNO-SRF) was introduced on top of the anode, such that NO_3_^–^ can be continuously replenished from the electrolyte-swelled polymer gel during Li deposition to maintain an appreciable local NO_3_^–^ concentration at the anode. Freestanding LNO-SRF was fabricated by blade-coating a mixture of LiNO_3_ and poly(vinylidene fluoride-co-hexafluoropropylene) (PVDF-HFP) in acetone on a rough metal surface, followed by peeling-off. It could also be coated on battery separators for large-scale applications, which is compatible to the state-of-art battery manufacturing process.

Figure 2c shows the typical scanning electron microscopy (SEM) image of LNO-SRF, revealing a highly porous structure likely caused by the phase inversion of PVDF-HFP during fabrication in the presence of hygroscopic LiNO_3_^38^. No discernable salt crystals/agglomerates were observed from high-magnification SEM image (Fig. 2d, Supplementary Fig. 6 shows the typical morphology of LiNO_3_ crystals), and uniform nitrogen distribution can be confirmed by energy-dispersive X-ray (EDX) elemental mapping (Fig. 2e–g). The high film porosity and the fine dispersion of LiNO_3_ species are particularly desirable for electrolyte wetting and rapid NO_3_^–^ release during battery operation. Thus, our method is far superior to directly adding LiNO_3_ solid into the electrolyte, which has been tested inefficacious for improving Li metal performance, due to inhomogeneous salt distribution (sedimentation) and slow dissolution kinetics of the large particles (Supplementary Fig. 7). The typical thickness of the membrane is ~18 μm, although much thinner film can also be prepared in practical batteries, and distinct LiNO_3_ peaks were observed from the X-ray diffraction (XRD) pattern, confirming the high mass loading of LiNO_3_ inside the LNO-SRF (Supplementary Fig. 8 and Supplementary Method 1; the particle size was calculated to be ~60 nm from the XRD data according to the Scherrer equation). The obtained LNO-SRF was dried inside an argon-filled glovebox with sub-ppm water and oxygen level at 120 °C for at least 1 week before use, in order to prevent the introduction of water contamination.

### High efficiency cycling in half- and full-cell configurations

**Fig. 3:**
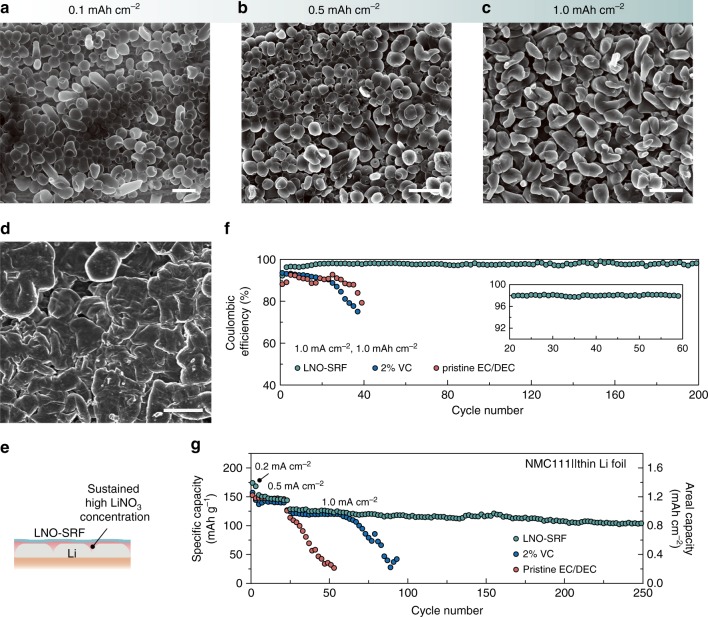
Deposition morphology and cycling CE with LNO-SRF. SEM images of the Li deposition morphology on Cu substrate covered by LNO-SRF at a current density of 1 mA cm^–2^ and a capacity of **a** 0.1 mAh cm^–2^, **b** 0.5 mAh cm^–2^, and **c** 1 mAh cm^–2^, respectively. **d** SEM image of the Li foil surface after 50 stripping/plating cycles in a Li||Li symmetric cell with LNO-SRF. The cycling was done at a current density of 1 mA cm^–2^ and a capacity of 1 mAh cm^–2^. Scale bars, 2 μm in **a** and 5 μm in **b**–**d**. **e** The solubility-mediated dissolution of LiNO_3_ from the microporous LNO-SRF can maintain a high local NO_3_^–^ concentration at the anode surface during battery cycling, which therefore results in a sustained modulation of Li deposition morphology. **f** Li cycling CE at a current density of 1 mA cm^–2^ and a capacity of 1 mAh cm^–2^ on Cu without additives (pristine EC/DEC), and with VC (2% VC) or nitrate (LNO-SRF) as additive. The electrolyte used in all cases were 0.5 M LiPF_6_ EC/DEC. **g** Long-term cycling performance of Li||NMC full-cell with a limited amount of excess Li (42 μm thin Li foil) in 0.5 M LiPF_6_ EC/DEC electrolyte (pristine EC/DEC) and in 0.5 M LiPF_6_ EC/DEC electrolyte with VC (2% VC) or nitrate (LNO-SRF) as additive. The areal mass loading of NMC was ~8 mg cm^–2^. The first two formation cycles were carried out at a current density of 0.2 mA cm^–2^, followed by 20 cycles at 0.5 mA cm^–2^, and the long-term cycling was at 1.0 mA cm^–2^

Figure 3a–c shows the morphological evolution of Li with increasing deposition capacities in the presence of LNO-SRF. Instead of growing into dendrites, as in the case of carbonates saturated with NO_3_^–^, the densely packed spherical Li nuclei evolved into nodule-like shapes with increasing capacity (Supplementary Fig.9). Moreover, the modulation effect can be preserved over repeated plating/stripping cycles on both Li foil (Fig. 3d) and Cu substrate (Supplementary Fig.10). Since the observation cannot be made using neat PVDF-HFP membrane alone (Supplementary Fig.11), such sustained control over Li deposition morphology shall be attributed to the fast, steady release of NO_3_^–^ from the microporous LNO-SRF, enabling a reasonable local additive concentration at the electroplating front despite its low solubility (Fig. 3e).

The Li anode cycling efficiency was determined by galvanostatic Li plating/stripping on Cu electrode at various current densities and capacities. Since reducing the LiPF_6_ concentration results in higher NO_3_^–^ solubility, 0.5 M LiPF_6_ in EC/DEC was chosen as the electrolyte. Further reducing the LiPF_6_ concentration was found to undermine the Li cycling efficiency (Supplementary Fig. 12). At a current density of 0.5 mA cm^–2^ and a capacity of 0.5 mAh cm^–2^, the CE on Cu electrode covered with LNO-SRF was indeed higher in 0.5 M LiPF_6_ EC/DEC electrolyte than in the 1.0 M counterpart, thanks to the increased additive concentration (Supplementary Fig. 13). Nevertheless, the reversibility of both cells was significantly better than those with bare Cu electrode or electrode covered with pure PVDF-HFP membrane, along with a much improved first-cycle CE (from ~85% on bare Cu to >92%; Supplementary Fig. 13).

Importantly, nitrate additive enabled by LNO-SRF considerably outperformed the conventional additives over extended cycling, promoting the Li cycling efficiency and stability in carbonate electrolytes to a level comparable to, if not better than, ether electrolytes (Fig. 3f and Supplementary Fig. 14). At a current density of 1 mA cm^–2^ and a capacity of 1 mAh cm^–2^, the LNO-SRF cell demonstrated high efficiency cycling stable over 200 cycles (averaged ~98.1% exclusive of the activation cycles), while the Li CE in EC/DEC electrolyte with 2 wt% VC started at ~93% and decayed rapidly within 40 cycles. Since additives such as FEC and VC fail to improve the deposition morphology, high-surface-area Li dendrites can lead to non-negligible side reactions and excess “dead lithium” formation in each plating/stripping cycle^39^, which account for the fast CE fading. On the other hand, nitrate additive results in nodule-like Li deposits with reduced surface area (and more stable SEI as will be discussed later), which is beneficial for reversible Li cycling. This can be further confirmed from the stable Li deposition polarization during prolonged cycling (Supplementary Fig. 15). Moreover, the solubility-mediated sustained release of NO_3_^–^ is generally applicable to carbonate-based electrolytes (Supplementary Fig. 16), and compromises neither the anodic stability nor the impedance of the cell (Supplementary Fig. 17), further corroborating the advantages of LNO-SRF.

To demonstrate that LNO-SRF in carbonate electrolytes can lead to more competitive Li metal batteries, we constructed full-cells with NMC as the cathode. Notably, only a finite amount of Li reservoir was employed in this study (either 42 μm Li foil or 150 μm three-dimensional Li metal-reduced graphene oxide composite anode, Li-rGO; Supplementary Fig. 18)^8^. Assuming the cathode has decent reversibility, the capacity fading of the full-cell shall be associated mainly to the loss of Li at the anode side (Supplementary Fig. 19). At a cathode mass loading of 8 mg cm^–2^, the cell cycled in pristine 0.5 M LiPF_6_ EC/DEC electrolyte exhibited an extremely limited reversibility, the capacity of which started to decay rapidly after merely 25 cycles due to the depletion of Li metal (Fig. 3g). The addition of 2% VC moderately improved the electrochemical performance of the full-cell, extending stable cycling to ~60 cycles. However, the life time of the Li||NMC cells can be more than quadrupled with the introduction of LNO-SRF, which demonstrated little capacity decay up to >250 cycles. Moreover, when the NMC cathode with commercial-level mass loading (~20 mg cm^–2^) was paired with three-dimensional Li anode (Li-rGO composite)^8^, the electrochemical cycling stability of the full-cell with LNO-SRF can even outperform the one with VC additive and nearly-infinite Li reservoir (Supplementary Fig. 20), further confirming the unique advantages of LNO-SRF over conventional additives in carbonate electrolytes. Admittedly, the reported cycling stability still falls short of the stringent requirements of commercial Li-ion batteries. Nevertheless, the effectiveness and practicality of our proposed solubility-mediated sustained nitrate release strategy in improving the safe and efficient operation of the Li metal anode is clear from the electrochemical results. We are positive that future improvements can be expected when LNO-SRF is used in conjunction with other strategies that have been proven efficacious for Li metal stabilization, such as nanostructuring the electrode^40^, and special electrochemical cycling protocols^41^.

## Discussion

**Fig. 4:**
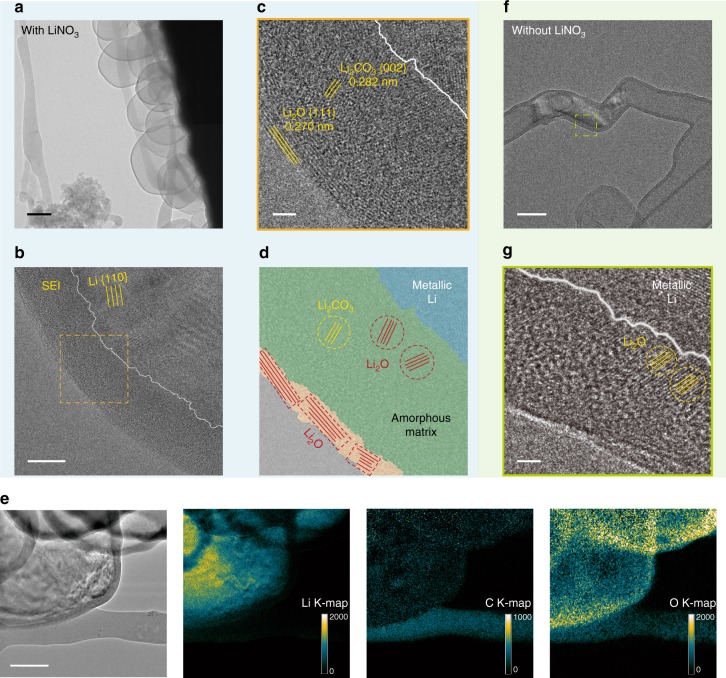
Cryo-EM study on the SEI structure in the presence of nitrate. **a** Low-magnification and **b** high-resolution TEM images of Li deposits in 0.5 M LiPF_6_ EC/DEC electrolyte saturated with NO_3_^–^ at a current density of 0.5 mA cm^–2^ for 150 s. The white line in **b** delineates the boundary between metallic Li and SEI. Scale bars, 200 nm in **a** and 10 nm in **b**. **c** Magnified TEM image and **d** the corresponding schematic of the orange region outlined in **b** showing the bilayered structure of SEI. The outside is a dense layer of Li_2_O displaying clear lattice fringes, and the inside is an amorphous matrix with crystalline domains (Li_2_O and Li_2_CO_3_) dispersed randomly throughout. Scale bar, 2 nm in **c**. **e** Energy-filtered TEM mapping of elemental distributions of Li, C, and O across the Li deposits and the corresponding unfiltered TEM image of the region. Scale bar, 100 nm. **f** Low-magnification and **g** high-resolution TEM images of Li deposits in 0.5 M LiPF_6_ EC/DEC electrolyte without NO_3_^–^. The SEI showed a mosaic model with crystalline domains dispersed in an amorphous matrix. Scale bars, 100 nm in **f** and 2 nm in **g**

To elucidate the mechanism behind the improved morphology and electrochemical performance of Li metal anode with nitrate additive, which is likely originated from the modified SEI properties, we first resolved the structure of the SEI via transmission electron microscopy (TEM) at cryogenic temperature^30^. The Cryo-EM technique was recently developed in our lab to successfully visualize fragile battery materials with atomic scale resolution at their pristine states without damage. Li was deposited on Cu TEM grid using standard battery condition (Supplementary Fig. 21) and loaded into TEM strictly following the cryo-transfer protocol without air exposure, such that the structural and chemical information of the SEI can be preserved at its native state. Figure 4a is a typical cryo-EM image of Li nuclei deposited in carbonate electrolyte with NO_3_^–^, and the edge of the particles with darker contrast corresponds to the SEI region (due to the higher atomic numbers of the SEI components than elemental Li). Lattice spacings matching the {110} planes of metallic Li can be clearly resolved (Fig. 4b), confirming the crystallinity of the electroplated Li. Importantly, high-resolution cryo-EM images of the SEI clearly revealed a bilayered structure (Fig. 4c, d and Supplementary Fig. 22). The outer layer consisted of highly ordered crystalline Li_2_O, while the inner layer appeared as an amorphous matrix with nanocrystallites dispersed inside (mainly Li_2_O and Li_2_CO_3_). Energy-filtered TEM images (Fig. 4e) confirmed the high concentration of Li at the inside of the deposits (metallic Li), the even distribution of carbon throughout the SEI (Li_2_CO_3_, alkyl carbonates, polymeric compounds from solvent decomposition, etc.), and the predominant localization of oxygen on the outside due to the dense Li_2_O shell. Although no crystalline phases containing nitrogen can be observed, nitrogen can indeed be detected over the SEI region via the electron energy loss spectroscopy (EELS, Supplementary Fig. 23). Thus, the nitrogen species from the preferential reduction of NO_3_^–^ shall remain amorphous in the SEI (or the size of their domains are too small to be observed by TEM possibly due to limited concentration). This bilayered SEI configuration stands a stark contrast to the mosaic model of SEI in pristine EC/DEC electrolyte reported previously^30^, where only the amorphous matrix with dispersed nanocrystals was seen (Fig. 4f, g).

One hypothesis to account for the formation of the distinct bilayered structure is that, during the SEI formation, NO_3_^–^ is reduced first to various insoluble N_*x*_O_*y*_^–^ species and Li_2_O^42,43^. The crystallization of Li_2_O is kinetically favorable, forming a dense crystalline layer^44^, while the N_*x*_O_*y*_^–^ species remain largely amorphous. However, this initial SEI layer cannot completely prevent the permeation of electrolyte given the volume change of Li metal during deposition, such that additional reactions between the solvents and Li result in the amorphous inner layer, until the electron tunneling limit is reached. This observation is in line with previous studies based on surface-sensitive techniques^45^.

The dense, uniform inorganic SEI outer layer can profoundly improve the homogeneity of the SEI, which not only promotes even Li-ion flux during deposition to suppress dendrites, but also effectively shields the Li surface from electrolyte corrosion to improve the cycling efficiency. Note that although FEC additive also results in a bilayered SEI structure as reported previously^30^, it does not form spherical Li nuclei. Therefore, the exact Li deposition morphology also relies heavily on the physiochemical properties (ion-transport kinetics, interfacial energy, etc.) of the amorphous components of the SEI, which is beyond the information that can be provided from cryo-EM.

**Fig. 5:**
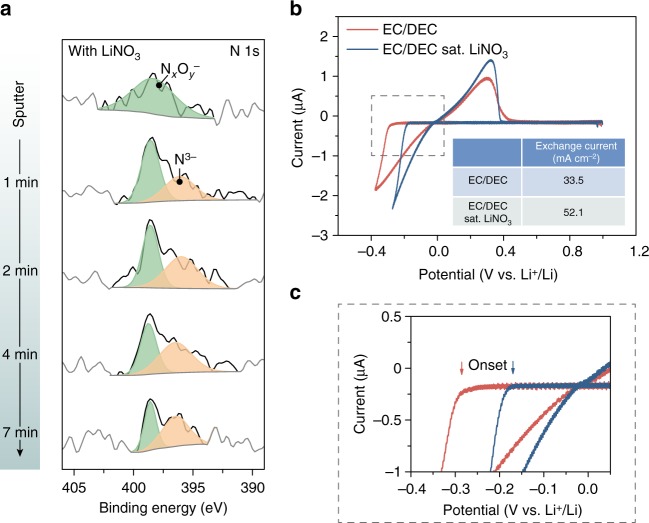
XPS and exchange current measurements. **a** XPS N_1s_ depth profiles of the SEI formed in 0.5 M LiPF_6_ EC/DEC electrolyte saturated with NO_3_^–^. Li deposition was carried out at a current density of 1 mA cm^–2^ and a capacity of 0.1 mAh cm^–2^ on Cu foil. **b** Typical CV scans (scan rate of 200 mV s^–1^) obtained using ultramicroelectrode in 1.0 M LiPF_6_ EC/DEC electrolyte with and without nitrate additive. **c** Enlarged CV scans around the nucleation potential region delineated in **b**. The presence of NO_3_^–^ in the electrolyte lowers the Li deposition onset potential by more than 100 mV

To correlate the formation of spherical Li with the chemical compositions of the SEI, XPS analysis with step-by-step sputtering was carried out on electrodeposited Li nuclei in EC/DEC electrolyte with nitrate additive. Consistent with the EELS data, XPS showed clear N_1s_ signal in the SEI (Fig. 5a). The surface nitrogen species possessed higher oxidation states (LiN_*x*_O_*y*_), while more Li_3_N can be detected closer to the Li side. Compare to the SEI formed in neat EC/DEC electrolyte, no other significant compositional differences can be detected in XPS, apart from slight concentration variations of different SEI components and a higher LiF content (Supplementary Figs. 24–27). Therefore, the nitrogen species from the preferential reduction of NO_3_^−^ can naturally be interpreted to have a profound effect on the Li surface chemistry, likely by changing the ion-transport property of the SEI, since Li_3_N has long been known to exhibit superior Li-ion conductivity^46^. Such speculation can be verified via EIS measurement on Li||Li symmetric cells (Supplementary Fig. 28). It was found that FEC and VC resulted in a more resistive SEI, possibly due to the high LiF content with limited Li-ion conductivity, and the cell impedance increased gradually with time^47^. However, the presence of NO_3_^–^ substantially reduced the impedance of Li electrodes and the values slightly decreased upon storage, which can be ascribed to the further reduction of LiN_*x*_O_*y*_ to Li_3_N. The low-resistance SEI layer would increase the probability of Li adatom insertion on the bulk surface, suppressing deposition on the tips/kinks of existing protrusions to obstruct dendrite formation^48^, which account for the unique spherical Li nuclei observed in the presence of nitrate additive in carbonate electrolytes.

Finally, the exchange current of the Li^+^/Li couple in carbonate electrolyte with nitrate additive was measured using ultramicroelectrode, in an effort to shed light on the improved electrochemical performance from the charge transfer kinetics perspective (Supplementary Method 2). Ultramicroelectrode (tungsten wire 25 μm in diameter) was employed such that Li can be freshly deposited and then immediately stripped at a rate fast enough to minimize the interference from the Li surface films, and high-quality data can be obtained without being distorted by the solution ohmic drop^49^. As calculated from the CV scans shown in Fig. 5b, the exchange current of Li^+^/Li in 1.0 M LiPF_6_ EC/DEC electrolyte was on average 33.5 mA cm^–2^, while the value increased by more than 50% when the electrolyte is saturated with NO_3_^–^ (52.1 mA cm^–2^). Moreover, with the addition of nitrate additive, a much lower (more than 100 mV) nucleation onset potential can be observed from the CV scans, further indicates the improved charge transfer kinetics (Fig. 5c).

The increased exchange current reinforces our conclusions from the EIS results and is in line with the observation of a more controlled Li deposition morphology with nitrate additive, since higher exchange current tends to result in larger-sized Li deposits. Specifically, metal electrodeposition on inert electrodes begins with the formation of separate growth centers, and once a nucleus has been formed, the passing current causes a local deformation of the electric field, resulting in a potential drop in its vicinity that prevent the occurrence of new nucleation events (i.e. the nucleation exclusion zone). High exchange current serves to increase the size of the nucleation exclusion zones, which can reduce the nucleation rate to form bigger Li particles with reduced surface area^50^. To mechanistically explain the enhanced exchange current measured by ultramicroelectrode, we speculate that, due to the high DN, nitrate anions can partially replace the solvent molecules in the Li^+^ solvation structure, and therefore substantially weakens the interaction strength between Li^+^ and solvents to facilitate ion desolvation, which is considered as the rate-limiting step in the charge transfer process^51^.

In conclusion, the physiochemical properties of the SEI layer, which largely rely on electrolyte composition, have a profound impact on the electrochemical cycling of metallic Li. In this study, we discuss the possibility of nitrate anions as an “electrolyte-agnostic” additive, which can be preferentially reduced during SEI formation to afford an interfacial environment conducive for controlled Li deposition, regardless of the electrolyte systems. It is discovered with surprise that NO_3_^–^ at millimolar-level in carbonate electrolytes can dramatically transform the morphology of Li nuclei from dendritic to spherical due to the significantly modified SEI properties. Structurally, a bilayered SEI can be observed with a dense inorganic shell, which effectively protects the Li metal surface and homogenizes the ion flux. Electrochemically, the nitrogen-containing species in the SEI result in improved ion-transport properties, and the presence of NO_3_^–^ in the Li-ion solvation sheath leads to faster charge transfer kinetics, both of which are favorable for containing dendrite growth. Moreover, a solubility-mediated sustained-release strategy is proposed to overcome the solubility limitation of nitrate salts in carbonates, and therefore enabling Li cycling efficiency and stability in corrosive carbonates on par with ether electrolytes. The distinct advantages of “electrolyte-agnostic” additive shed new light on electrolyte engineering principles without compromising the ionic conductivity, stability, and cost of the electrolyte, and can open up opportunities to the realization of high-energy-density batteries based on Li metal chemistries.

## Methods

### Fabrication of the LNO-SRF

In all, 10 wt% PVDF-HFP (Kynar Flex 2801–00) and 5 wt% LiNO_3_ (Alfa Aesar, approximately the maximum solubility of LiNO_3_ in acetone) were fully dissolved in acetone by vigorous magnetic stirring on a 60 °C hotplate. The solution was then blade-coated on the rough side of electrodeposited Cu foil (CF-T8G-UN; Pred. Materials International, Inc.) using a 15-mil blade. Once the solvent was evaporated, the white-colored semi-transparent film can be peeled off from the Cu substrate. The obtained LNO-SRF was punched into 2 cm^2^ round disks and transferred quickly into an argon-filled glovebox with sub-ppm O_2_ and H_2_O level (Vigor Tech) to minimize water absorption. The LNO-SRF was dried on a 120 °C hotplate inside the glovebox for at least 1 week prior to use to prevent water contamination inside the battery.

### Electrochemical measurements

1.0 M LiPF_6_ in EC/DEC electrolyte was purchased from BASF (Selectilyte LP40). 0.1 and 0.5 M LiPF_6_ in EC/DEC electrolyte was made by diluting Selectilyte LP40 with EC (Acros Organics) and DEC (Acros Organics) solvents. LiTFSI (Solvay), LiFSI (Nippon Shokubai), DME (Sigma-Aldrich), FEC (Acros Organics), and VC (Acros Organics) were used as received. All the solvents were dried over 4A molecular sieves (Sigma-Aldrich) prior to making electrolytes.

Electrochemical testing was all carried out in 2032-type coin cell configuration. For studying the Li deposition morphology and cycling CE, the LNO-SRF was covered on top of the anode Cu current collector (25 μm, 99.8% metals basis; Alfa Aesar). Li foil (750 μm; Alfa Aesar) was used as the counter/reference electrode, two layers of Celgard 2325 (25 μm PP/PE/PP) was used as separators, and 80 μl electrolyte was added in each coin cell. The cells were rested for 3 h before testing to allow the LNO-SRF to fully swell in the electrolyte. For measuring the CE, the batteries were first subjected to 10 activation cycles between 0 and 1.5 V to form a stable SEI and eliminate side reactions before galvanostatic Li plating/stripping at different current densities and capacities on a LAND 8-channel battery tester. The LNO-SRF cells were rested 1 h between each plating/stripping cycle. For the Li||NMC full-cell studies, either thin Li foil (~50 μm, Hydro-Québec) or Li-rGO was employed as the anode^8^, and LNO-SRF was covered on the anode surface. The cathode was prepared by mixing LiNi_1/3_Mn_1/3_Co_1/3_O_2_ (NMC111, Xiamen Tungsten Co.), polyvinylidene fluoride (MTI), and carbon black (TIMCAL) at a ratio of 9:0.5:0.5 with *N*-methyl-2-pyrrolidone (Sigma-Aldrich) as the solvent. The cathode slurry was blade-coated on conductive carbon coated aluminum foil (MTI), calendared, and dried in 80 °C vacuum oven before use. All the Li||NMC batteries were tested between 2.7 and 4.3 V. When the voltage reached 4.3 V, a constant voltage charge process (4.3 V) was applied until the charge current decayed to 0.1 mA cm^–2^. The EIS, CV, and exchange current density measurements were carried out on a Biologic VMP3 system. Detailed descriptions on the exchange current density measurements are provided in Supplementary Information.

### Characterization

SEM images and EDX elemental mappings were taken with a FEI XL30 Sirion scanning electron microscope at an acceleration voltage of 5 and 20 kV, respectively. Before conducting SEM studies of Li electrodes, batteries were first disassembled in an argon-filled glovebox with sub-ppm O_2_ and H_2_O level, and then rinsed gently with DEC to remove residue Li salts. The samples were transferred into SEM with minimal air exposure. XRD patterns were recorded on a PANalytical X’Pert instrument. XPS analysis was obtained on a PHI VersaProbe 1 scanning XPS microprobe equipped with Al (Kα) source and argon ion sputter gun with an air-free transfer vessel. The binding energies were calibrated with respect to the C_1s_ peak at 284.5 eV. Argon sputtering for the XPS depth-profiling was carried out at a beam energy of 1 kV and current of 0.5 μA over a raster area of 2 mm × 2 mm. The sputtering rate calibrated on SiO_2_ surface was ~2 nm min^–1^.

Nitrate solubilities in different carbonate electrolytes were determined using a WestCo SmartChem 200 discrete analyzer. In a typical experiment, 200 μl electrolyte saturated with LiNO_3_ was diluted to 5 ml with water. During analysis, nitrate anions in the solution were reduced quantitatively to nitrite by a cadmium column, diazotized with sulfanilamide, and subsequently coupled with *N*-(1-naphthyl)ethylenediamine dihydrochloride. The resulting colored azo dye was colorimetrically detected at 550 nm.

Cryo-EM characterizations were carried out on a FEI Titan 80–300 environmental (scanning) transmission electron microscope (E(S)TEM) operated at 300 kV following the cryo-transfer protocol described in our previous publication^29^.

## Electronic supplementary material

Supplementary Information

Peer Review File

## Data Availability

The datasets generated during and/or analyzed during the current study are available from the corresponding author on reasonable request.
