# Supplementary material for: Solubility-mediated sustained release enabling nitrate additive in carbonate electrolytes for stable lithium metal anode
Source: Nat Commun. 2018 Sep 7;9:3656. doi: 10.1038/s41467-018-06077-5 (PMC6128910; doi:10.1038/s41467-018-06077-5)
Supplement: Supplementary file 1 — Supplementary Information [file 41467_2018_6077_MOESM1_ESM.pdf]

Supplementary Information

Solubility-mediated sustained release enabling nitrate additive in carbonate electrolytes for stable lithium metal anode

Liu et al

Supplementary Figures

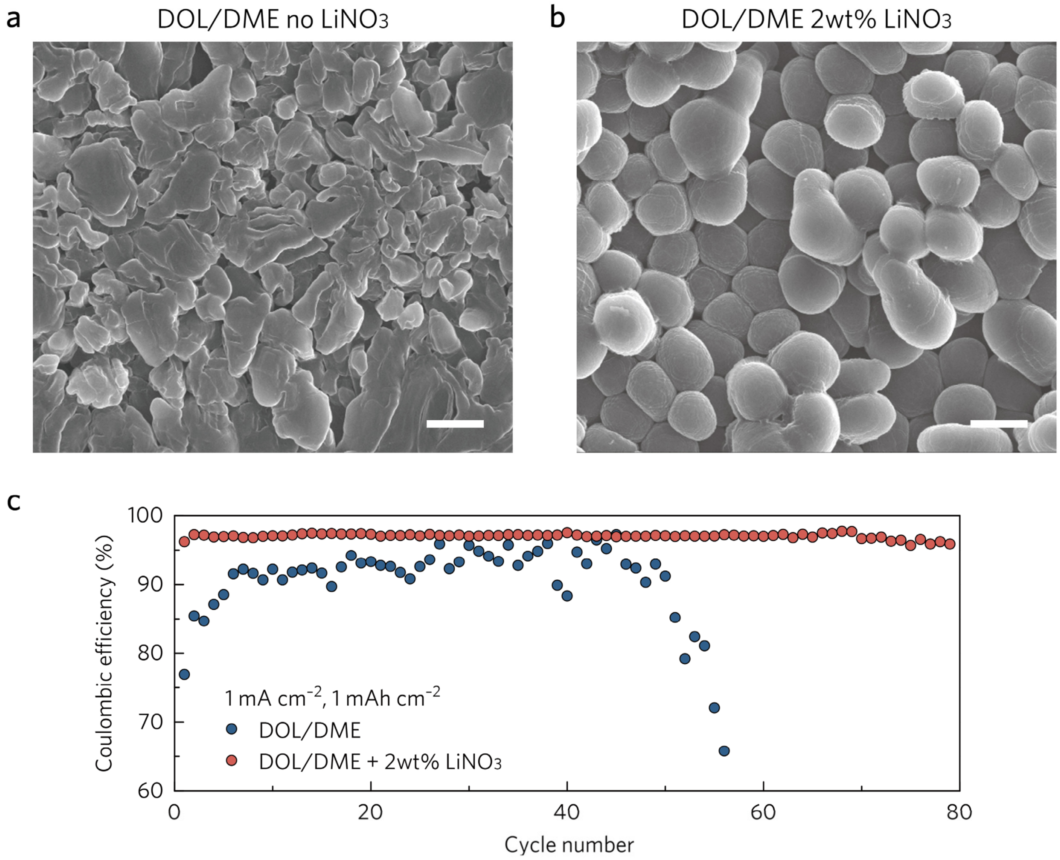

Supplementary Figure 1. The effect of nitrate on Li deposition morphology and CE in ether electrolyte. SEM images of Li deposition morphology using 1.0 M LiTFSI in 1:1 v/v 1,3-dioxolane/dimethoxyethane (DOL/DME) electrolyte (a) without and (b) with 2wt% $LiNO_3$ additive. The deposition was carried out at a current density of 1 mA cm^{-2} and a capacity of 0.5 mAh cm^{-2}. Scale bars, 2 μm. (c) Li metal cycling CE with and without $LiNO_3$ in DOL/DME electrolyte at a current density of 1 mA cm^{-2} and a capacity of 1 mAh cm^{-2}.

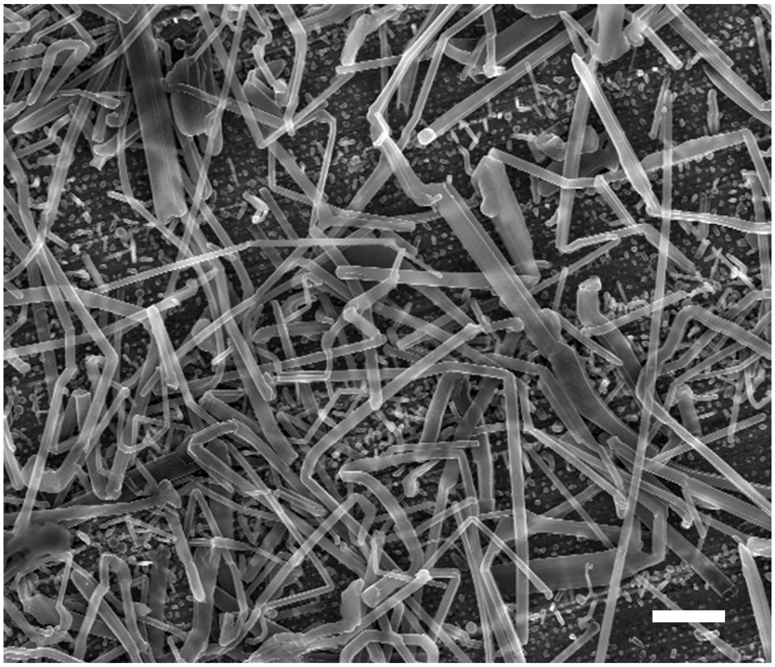

Supplementary Figure 2. SEM image of the Li deposition morphology in 1.0 M $LiPF_6$ EC/DEC electrolyte with 10wt% FEC and 2wt% VC as additives. The Li nuclei remained dendritic. The deposition was carried out at a current density of 1 mA cm^{-2} and a capacity of 0.1 mAh cm^{-2}. Scale bar, 2 μm.

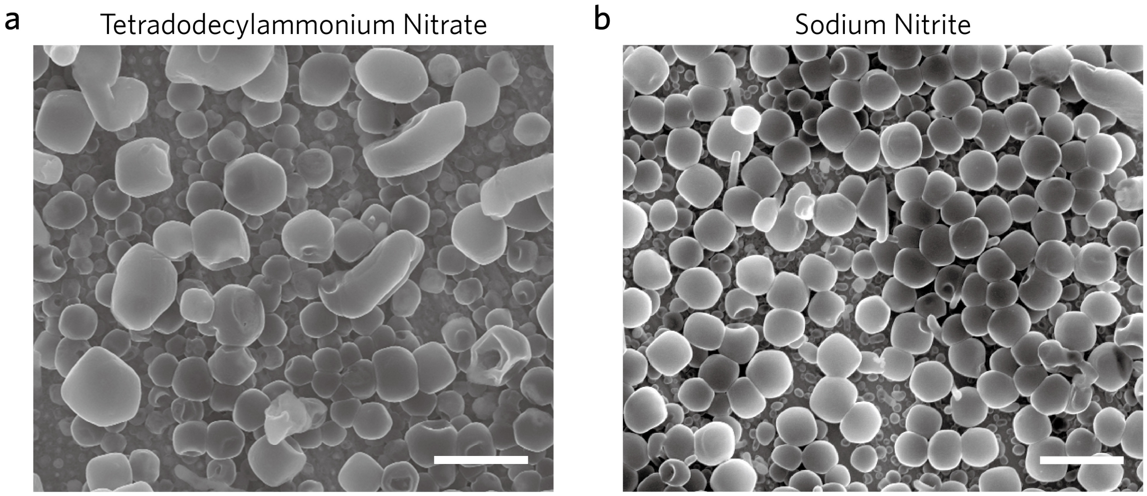

Supplementary Figure 3. The effect of nitrate on Li deposition morphology is indifference to the selection of cations and can be extended to other molecular species with N–O bond. SEM image of the Li deposition morphology in 1.0 M $LiPF_6$ EC/DEC electrolyte saturated with (a) tetradodecylammonium nitrate, and (b) sodium nitrite. The deposition was carried out at a current density of 1 mA cm^{-2} and a capacity of 0.1 mAh cm^{-2}. The Li nuclei were also spherical, therefore, additives with N–O bonds are generally effective in controlling Li morphology in carbonate electrolytes. Scale bars, 1 μm in (a) and 2 μm in (b).

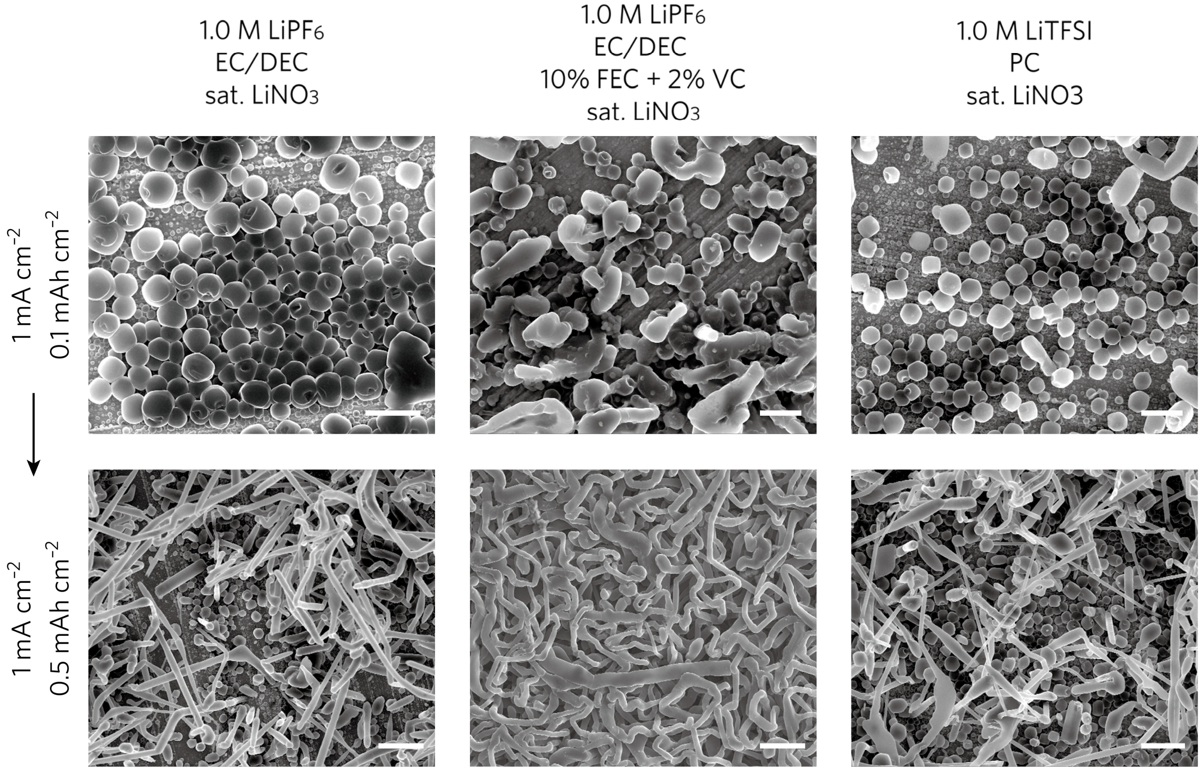

Supplementary Figure 4. SEM images of the Li deposition morphology in various carbonate electrolyte systems saturated with $LiNO_3$, including 1.0 M $LiPF_6$ in EC/DEC electrolyte, 1.0 M $LiPF_6$ in EC/DEC electrolyte with 10wt% FEC and 2wt% VC as additives, and 1.0 M LiTFSI in PC electrolyte. The depositions were carried out at a current density of 1 mA cm^{-2} and a capacity of either 0.1 mAh cm^{-2} (top row) or 0.5 mAh cm^{-2} (bottom row). Since nitrate anions are being continuously consumed to participate in the SEI formation during Li plating, its capability to control the Li morphology wears off quickly with increased deposition capacity, due to the extremely low solubility of nitrate in carbonate solvents. Scale bars, 2 μm for the top row, and 5 μm for the bottom row.

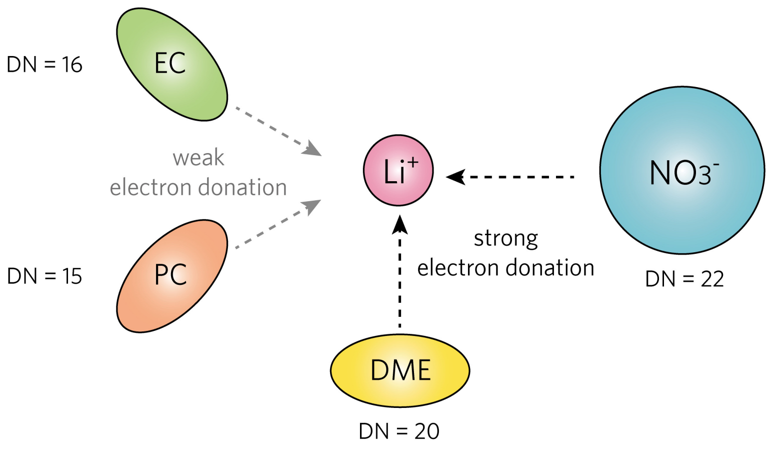

Supplementary Figure 5. Illustration of electron DN concept of Gutmann. For salt dissociation to occur, the DN of the solvent has to exceed the electronic interaction between the cation and anion itself. However, the DN value of NO_3^- (~22) is much higher than those of carbonates, accounting for the low solubility of nitrate salts in carbonates.

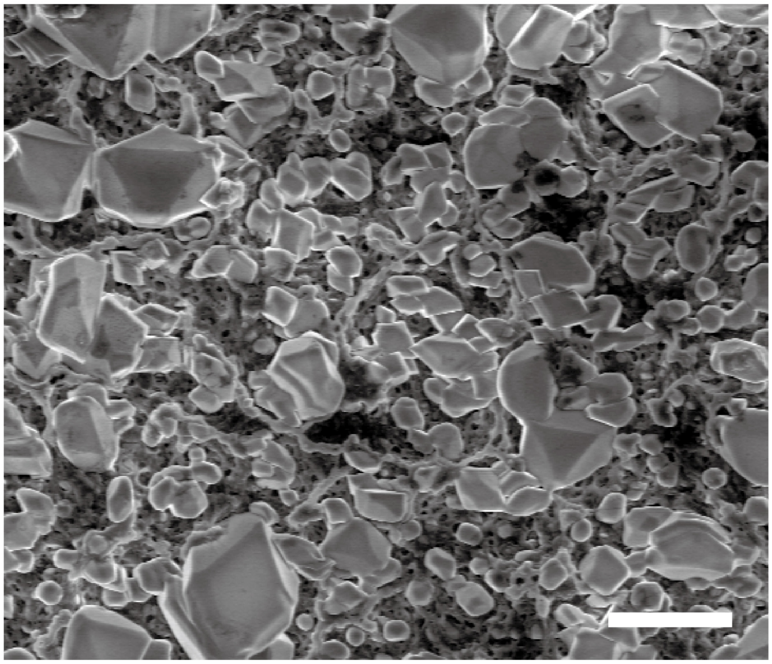

Supplementary Figure 6. SEM image of typical $LiNO_3$ crystals. The particles were obtained by evaporating 10wt% $LiNO_3$ dissolved in 9:1 v/v acetone/methanol. Scale bar, 5 μm.

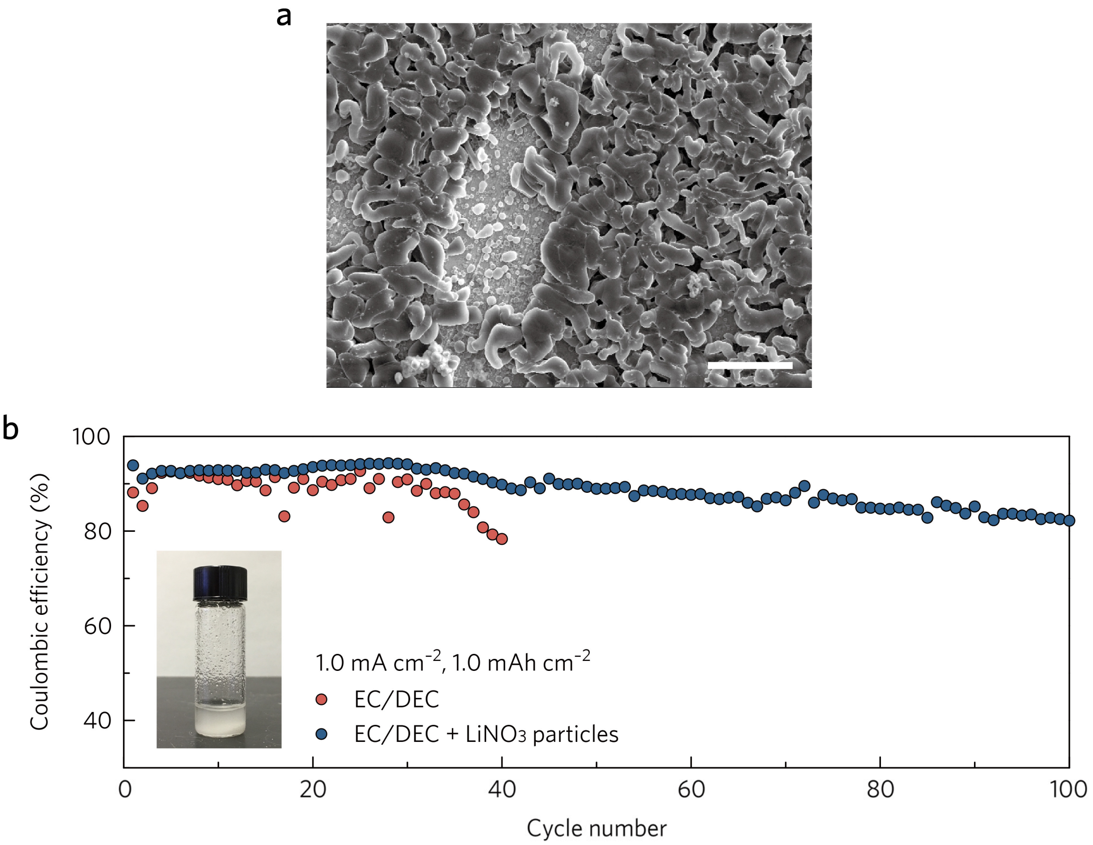

Supplementary Figure 7. Li deposition morphology and CE in carbonate electrolyte with the addition of fine-grained $LiNO_3$ particles. (a) SEM image of Li deposition using 1.0 M $LiPF_6$ in EC/DEC electrolyte with the addition of 2 wt% fine-grained $LiNO_3$ particles. Scale bar, 10 µm. (b) Li cycling CE on bare Cu using 1.0 M $LiPF_6$ in EC/DEC electrolyte with or without the addition of 2 wt% fine-grained $LiNO_3$ particles. Inset is the photo image of the electrolyte with $LiNO_3$ particles, where the electrolyte became cloudy with $LiNO_3$ sediments. The deposition and galvanostatic cycling were carried out at a current density of 1 mA cm^{-2} and a capacity of 1 mAh cm^{-2}. Due to the sedimentation and slow NO_3^- release kinetics of the large salt particles, the deposition remained dendritic and the CE only improved slightly compare to neat electrolyte.

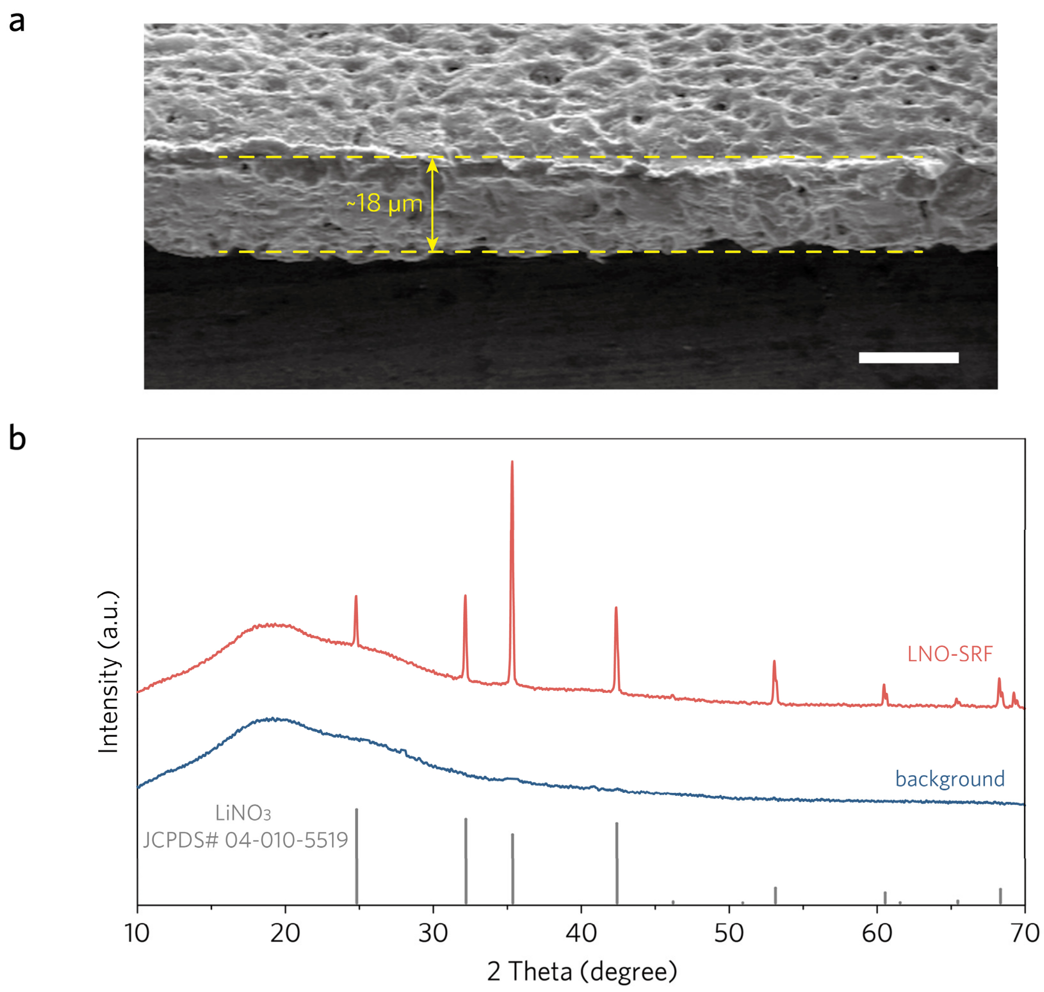

Supplementary Figure 8. Material characterizations of the LNO-SRF. (a) Cross-sectional SEM image of the LNO-SRF, indicating the thickness of the film to be ~18 μm. Scale bar, 20 μm. (b) XRD spectrum of the LNO-SRF, from which the particle size was calculated to be ~60 nm according to the Scherrer equation.

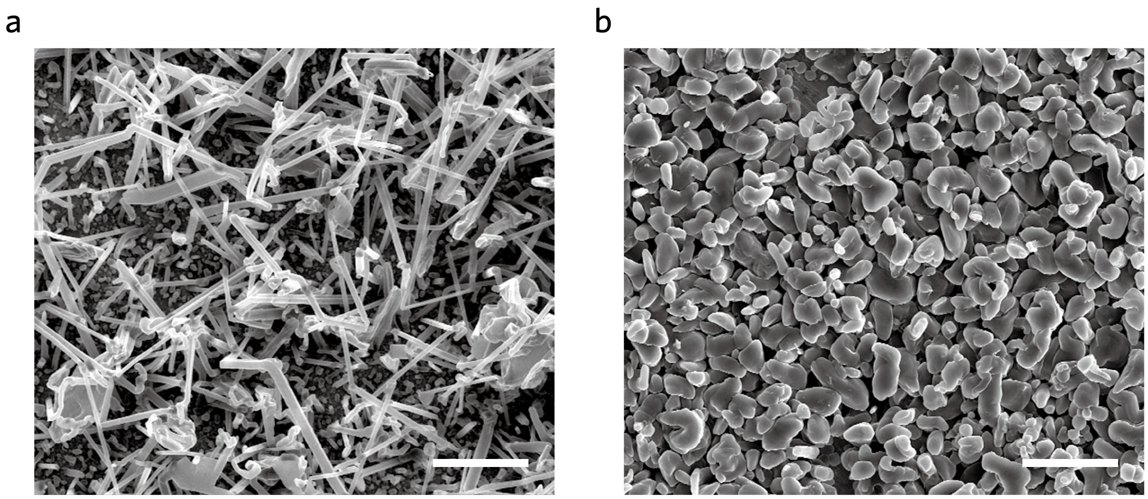

Supplementary Figure 9. Li deposition morphology with and without LNO-SRF. Low magnification SEM images of Li deposition at a current density of 1 mA cm^{-2} and a capacity of 1 mAh cm^{-2} in 0.5 M $LiPF_6$ EC/DEC electrolyte (a) on bare Cu, and (b) on Cu covered with LNO-SRF. Scale bars, 10 μm.

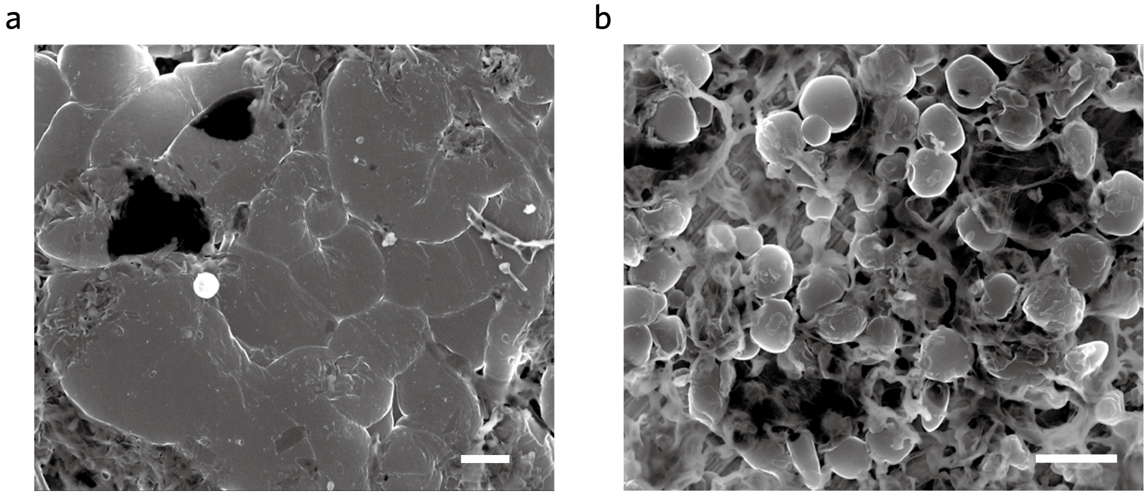

Supplementary Figure 10. SEM images showing the Li plating morphology on Cu foil substrate covered with LNO-SRF at the 10th deposition. (a) Dense Li region showing large, nodule-shaped deposit. Scale bar, 1 µm. (b) Sparse Li region near the edge of the Cu substrate, showing that the Li nuclei remained spherical even after repeated cycling. Scale bar, 2 µm. The cycling was carried out at a current density of 1 mA cm^{-2} and a capacity of 1 mAh cm^{-2} in 0.5 M $LiPF_6$ EC/DEC.

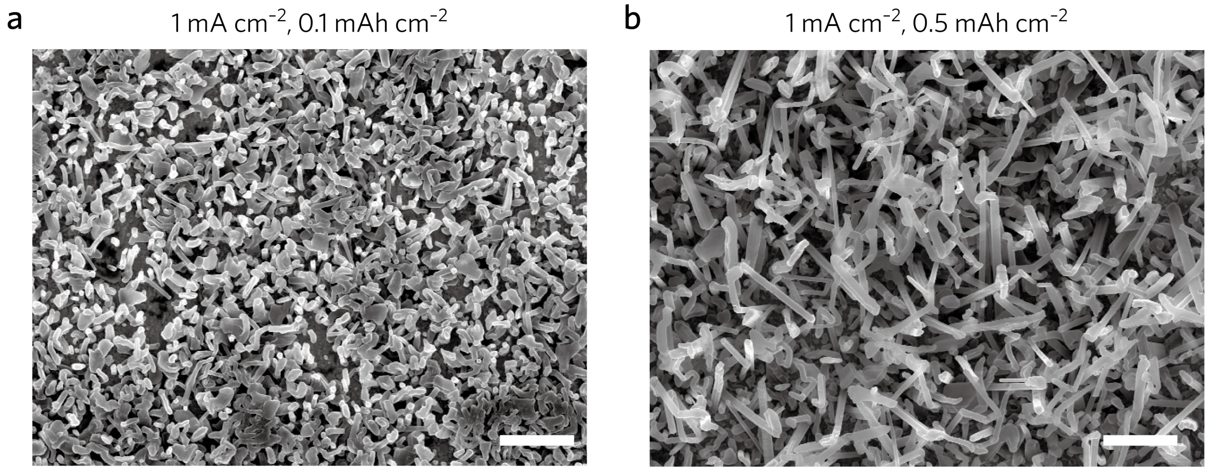

Supplementary Figure 11. Li deposition morphology under pure PVDF-HFP membrane of similar thickness as LNO-SRF in 0.5 M $LiPF_6$ EC/DEC electrolyte. The deposition was carried out at a current density of 1 mA cm^{-2} and a capacity of (a) 0.1 mAh cm^{-2}, and (b) 0.5 mAh cm^{-2}, respectively. Scale bars, 5 μm.

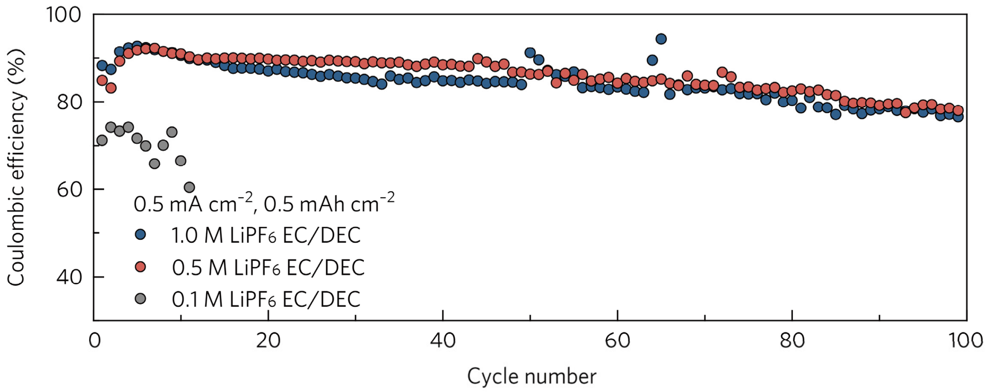

Supplementary Figure 12. Li cycling CE on bare Cu at a current density of 0.5 mA cm^{-2} and a capacity of 0.5 mAh cm^{-2} in EC/DEC electrolytes with different $LiPF_6$ concentrations. Reducing the $LiPF_6$ concentration from 1.0 M to 0.5 M showed no obvious effect on the electrochemical performance, while further reducing the concentration to 0.1 M negatively impacted the CE.

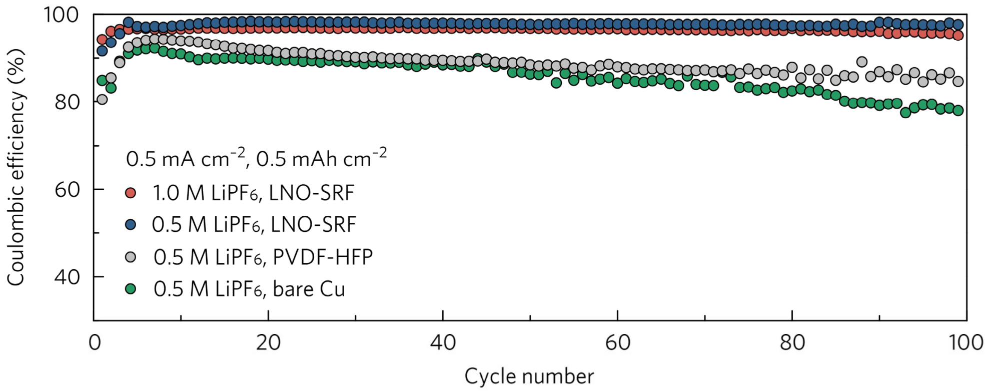

Supplementary Figure 13. Li cycling CE at a current density of 0.5 mA cm^{-2} and a capacity of 0.5 mAh cm^{-2} in EC/DEC electrolytes on Cu electrode covered with LNO-SRF, Cu electrode covered with neat PVDF-HFP membrane, and bare Cu electrode.

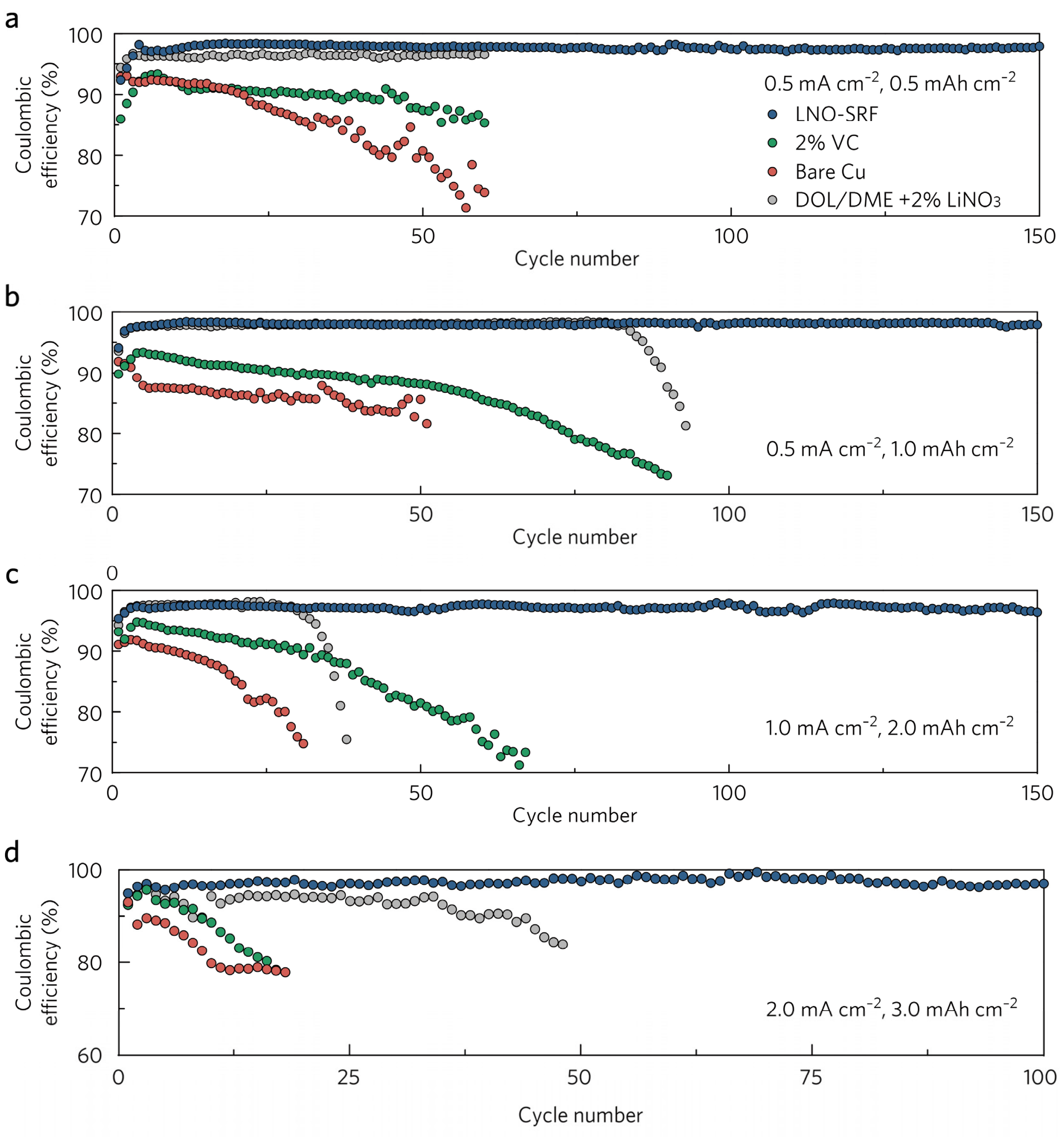

Supplementary Figure 14. Long-term Li cycling CE on Cu electrode in 0.5 M $LiPF_6$ EC/DEC electrolyte without additives (Bare Cu), and with VC (2% VC) or nitrate additives (LNO-SRF), or in 1.0 M LiTFSI DOL/DME electrolyte with 2wt% $LiNO_3$ additive (DOL/DME + 2% $LiNO_3$). (a) 0.5 mA cm^{-2} current density and 0.5 mAh cm^{-2} capacity, (b) 0.5 mA cm^{-2} current density and 1 mAh cm^{-2} capacity, (c) 1 mA cm^{-2} current density and 2 mAh cm^{-2} capacity, and (d) 2 mA cm^{-2} current density and 3 mAh cm^{-2} capacity. The average CE of LNO-SRF exclusive of the first ten activation cycles was 98.0%, 98.2%, 97.5% and 97.6%, respectively, which is comparable to, if not better than, the values in ether electrolyte (DOL/DME + 2% $LiNO_3$).

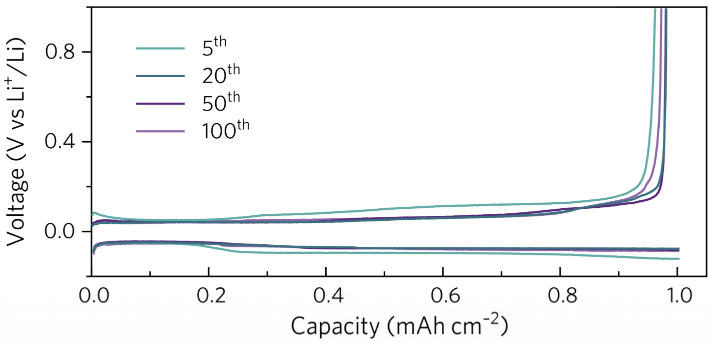

Supplementary Figure 15. Li plating/stripping profiles with LNO-SRF in 0.5 M $LiPF_6$ EC/DEC electrolyte at a current density of 1 mA cm^{-2} and a capacity of 1 mAh cm^{-2}.

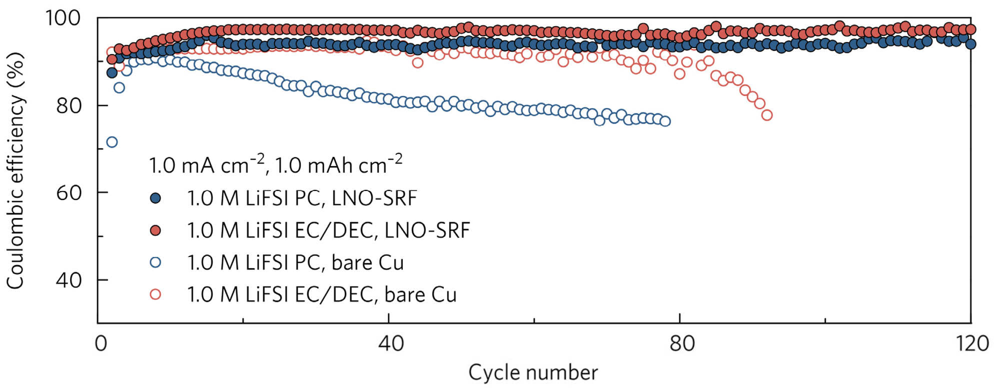

Supplementary Figure 16. Li cycling CE at a current density of 1 mA cm^{-2} and a capacity of 1 mAh cm^{-2} in different carbonate-based electrolyte systems with and without LNO-SRF. The LNO-SRF strategy can be generally applied in carbonate electrolytes to appreciably improve the cycling efficiency and stability.

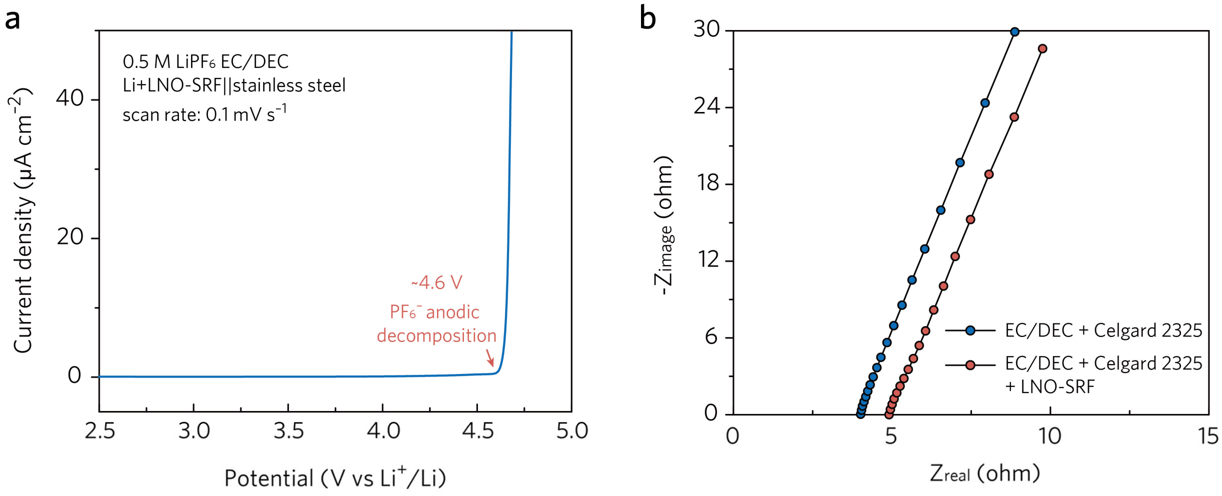

Supplementary Figure 17. The solubility-mediated sustained release approach compromises neither the stability nor the impedance of the battery. (a) Oxidation potential of 0.5 M $LiPF_6$ EC/DEC electrolyte with LNO-SRF by linear sweep voltammetry. (working electrode: stainless steel, counter/reference electrodes: Li foil covered with LNO-SRF, scanning rate: 0.1 mV s^{-1}). Anodic decomposition starts at ~4.6 V vs Li^+/Li due to $LiPF_6$ decomposition. Therefore, the adoption of LNO-SRF in carbonate-based electrolytes does not compromise the advantageous stability window of the electrolyte. (b) EIS spectra of coin cells with and without LNO-SRF. The measurement was done by sandwiching two layers of 2 cm^2 Celgard 2325 separators between two stainless steel spacers, and 50 μL of 0.5 M $LiPF_6$ in EC/DEC electrolyte was used in each coin cells. The additional LNO-SRF layer resulted in minimal increase in cell impedance.

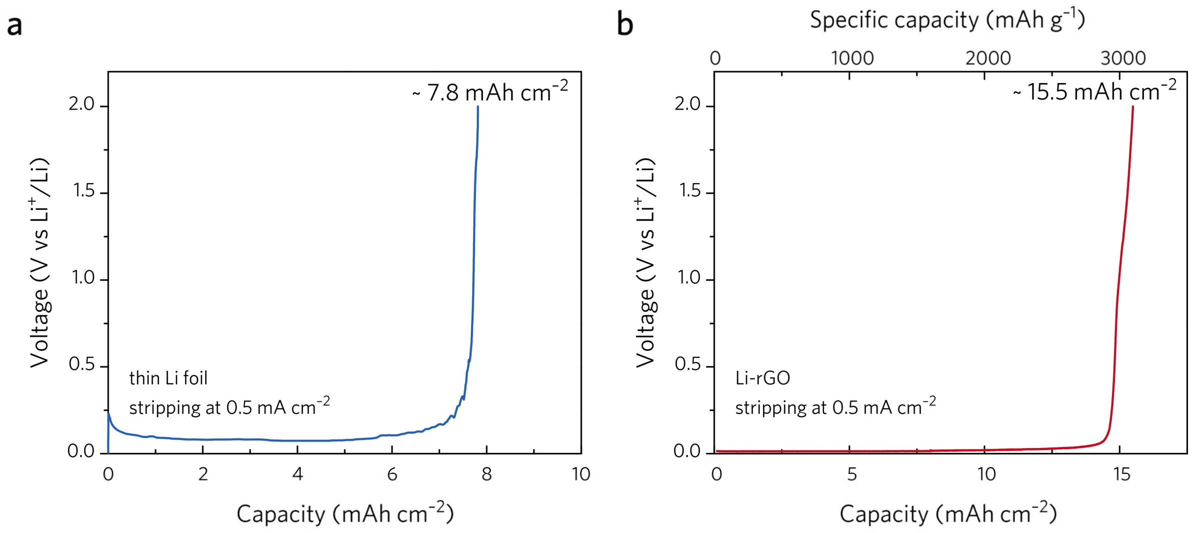

Supplementary Figure 18. Areal capacity of the thin Li foil and Li-rGO anode used in this study. (a) Full Li stripping curve of the thin Li foil (~42 μm) used in Li||NMC full-cells. The stripping capacity at a current density of 0.5 mA cm^{-2} in 0.5 M $LiPF_6$ EC/DEC electrolyte was ~7.8 mAh cm^{-2}. (b) Full Li stripping curve of the porous Li-rGO composite anode (~150 μm) used in Li||NMC full-cells. The stripping capacity at a current density of 0.5 mA cm^{-2} in 0.5 M $LiPF_6$ EC/DEC electrolyte was ~15.5 mAh cm^{-2}.

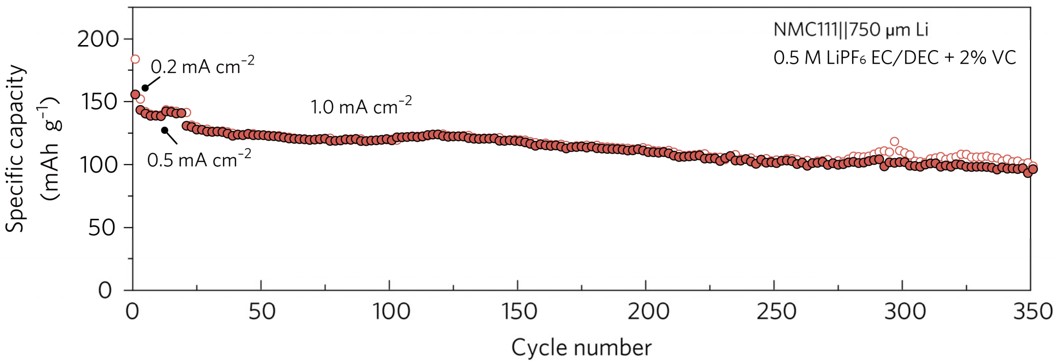

Supplementary Figure 19. Long-term cycling performance of NMC||Li full-cell with large amount of excess Li (750 μm Li foil) in 0.5 M $LiPF_6$ EC/DEC electrolyte with 2% VC additive. The areal mass loading of NMC was ~8 mg cm^{-2}. The first two formation cycles were carried out at a current density of 0.2 mA cm^{-2}, followed by 20 cycles at 0.5 mA cm^{-2}, and the long-term cycling was at 1.0 mA cm^{-2}.

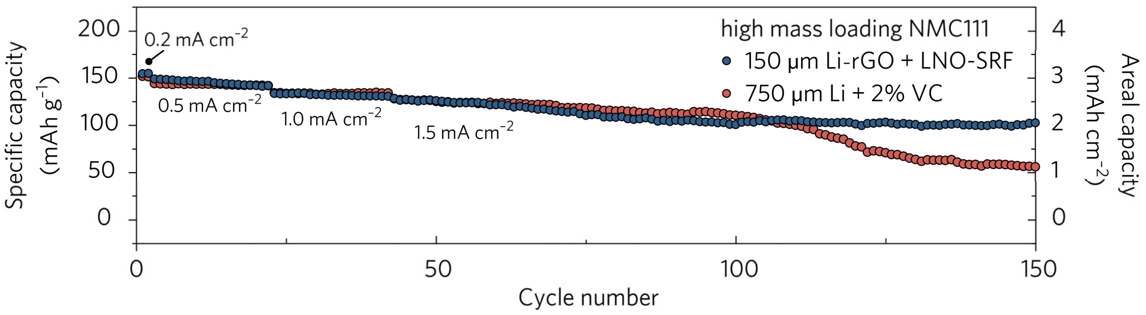

Supplementary Figure 20. Long-term cycling performance of NMC||Li full-cell with high mass loading cathode (~20 mg cm^{-2}). The red data points correspond to NMC cycled with a large amount of excess Li (750 μm Li foil) in 0.5 M $LiPF_6$ EC/DEC electrolyte with 2% VC additive. The blue data points correspond to NMC cycled with limited excess Li (150 μm Li-rGO composite anode) in the presence of LNO-SRF in 0.5 M $LiPF_6$ EC/DEC. The first two formation cycles were carried out at a current density of 0.2 mA cm^{-2}, followed by 20 cycles at 0.5 mA cm^{-2}, 20 cycles at 1.0 mA cm^{-2}, and the long-term cycling was at 1.5 mA cm^{-2}.

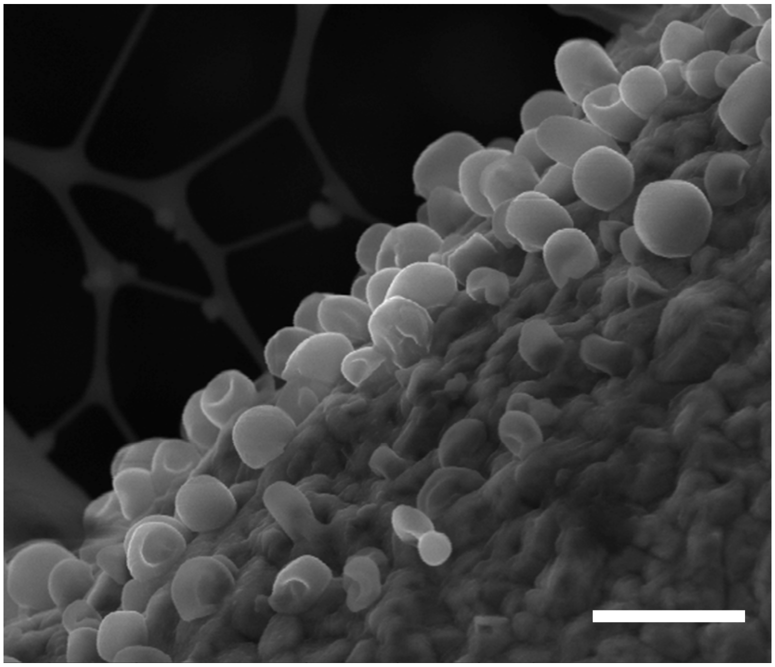

Supplementary Figure 21. SEM image showing Li deposition on Cu TEM grid for cryo-EM characterizations. The deposition was carried out in 0.5 M $LiPF_6$ EC/DEC electrolyte saturated with nitrate at a current density of 0.5 mA cm^{-2} for 150 s. Small deposition capacity was used to obtain small Li nuclei suitable for TEM observations and to prevent the depletion of nitrate in the electrolyte. Scale bar, 1 μm.

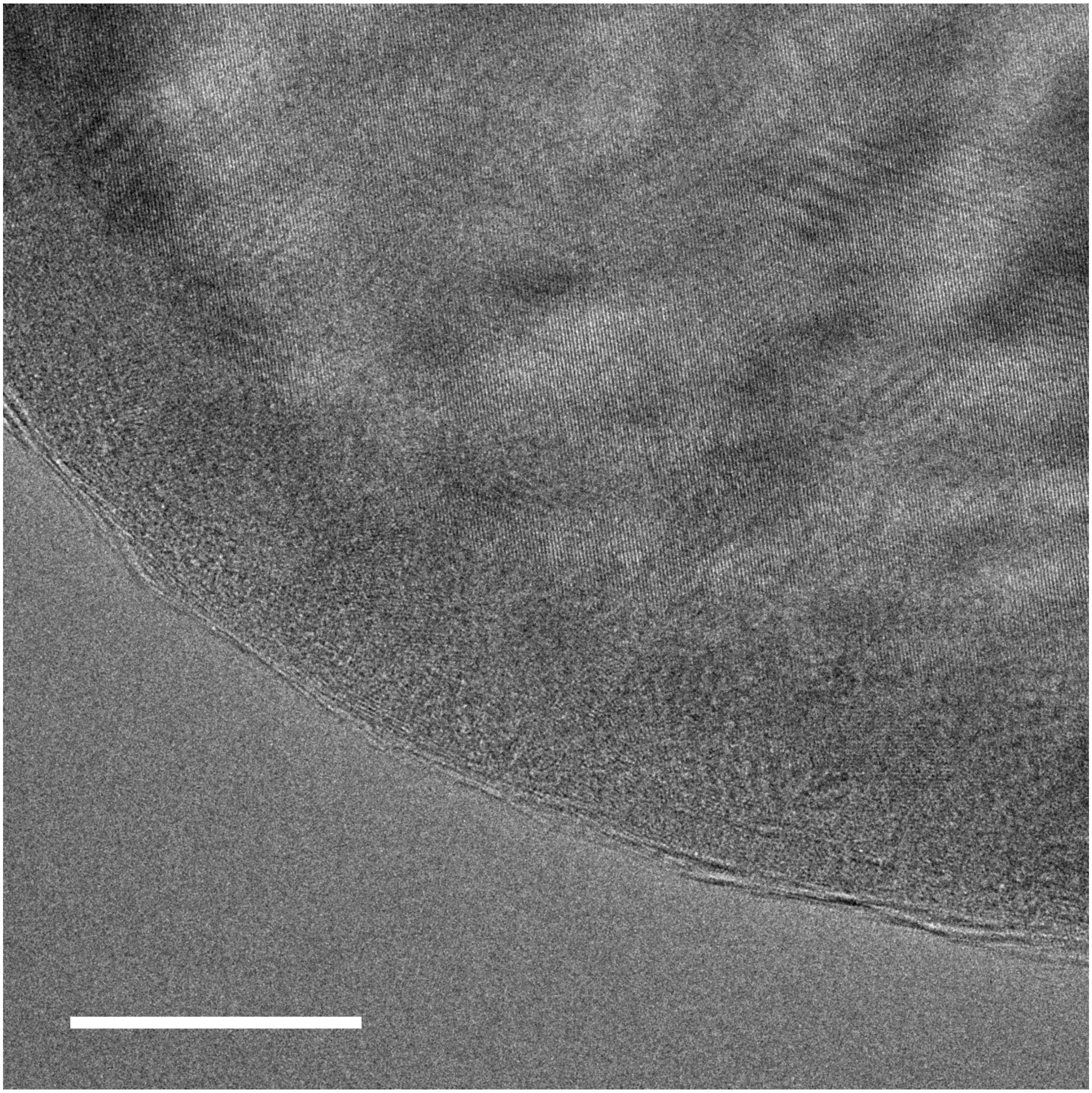

Supplementary Figure 22. Raw high-resolution cryo-EM image of the Li metal deposit in 0.5 M $LiPF_6$ EC/DEC electrolyte with nitrate additive. Scale bar, 20 nm.

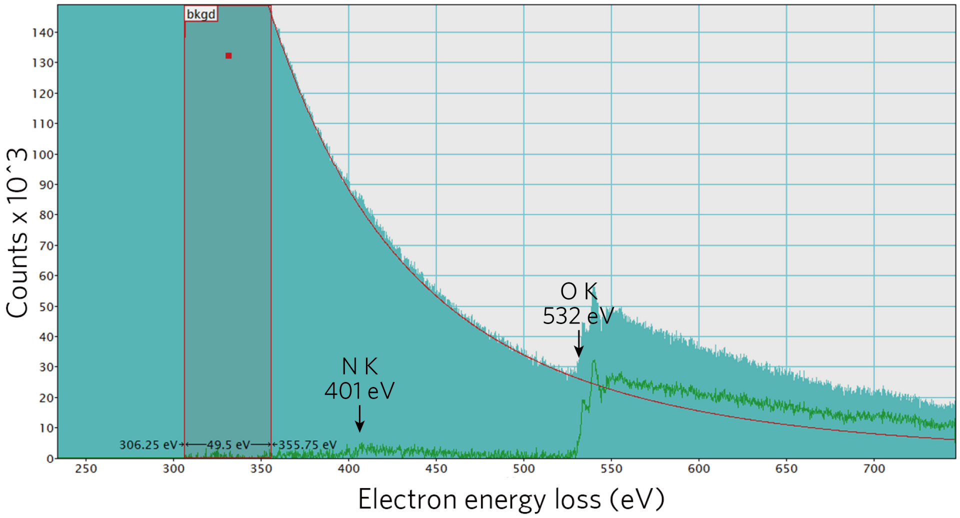

Supplementary Figure 23. EELS spectrum of the SEI formed on Li metal surface in 0.5 M $LiPF_6$ EC/DEC electrolyte with nitrate additive, where nitrogen content in the SEI can be detected.

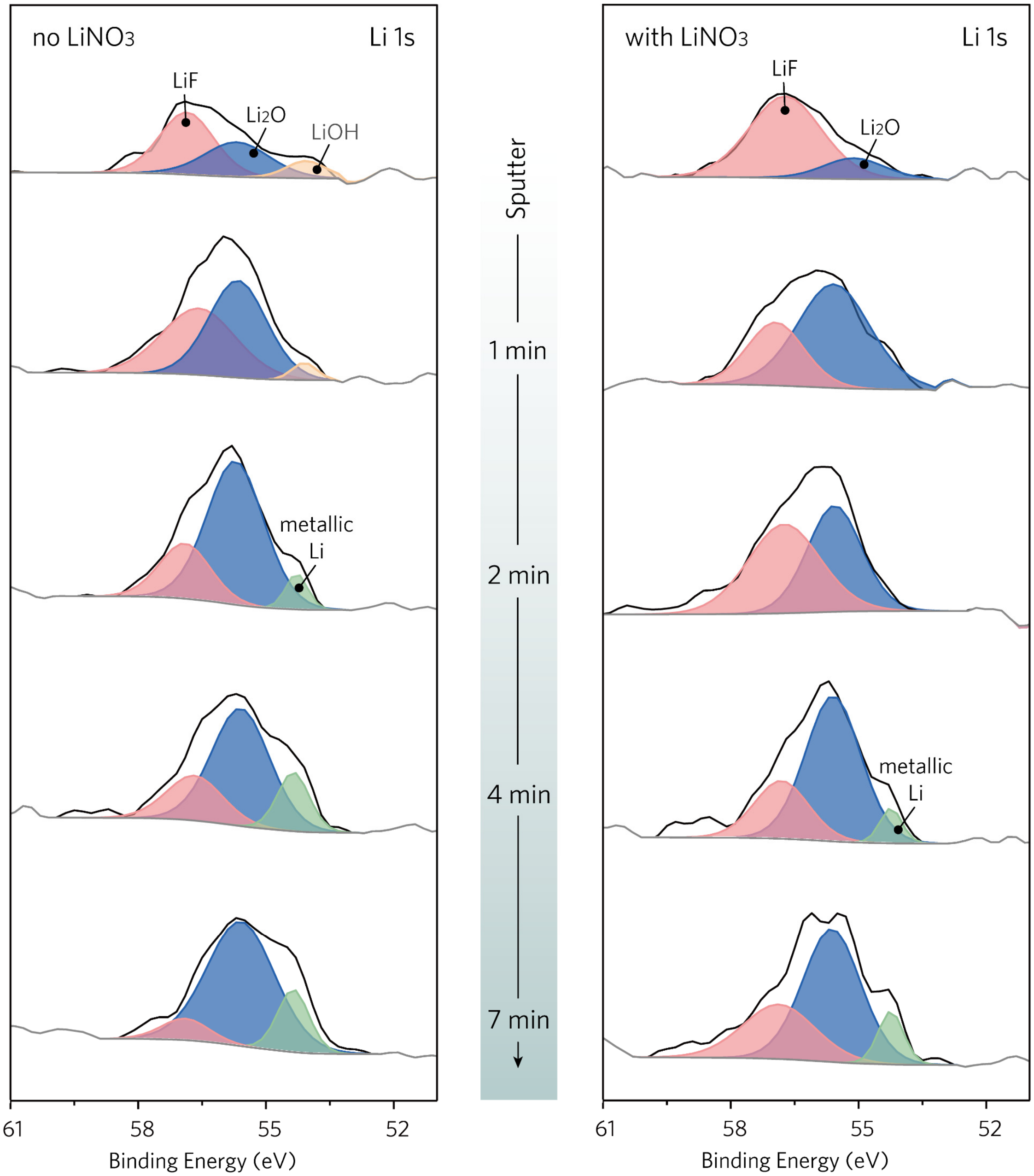

Supplementary Figure 24. XPS Li_{1s} depth profiles of the SEI formed in 0.5 M $LiPF_6$ EC/DEC electrolyte with or without nitrate additive. Li deposition was carried out at a current density of 1 mA cm^{-2} and a capacity of 0.1 mAh cm^{-2} on Cu foil.

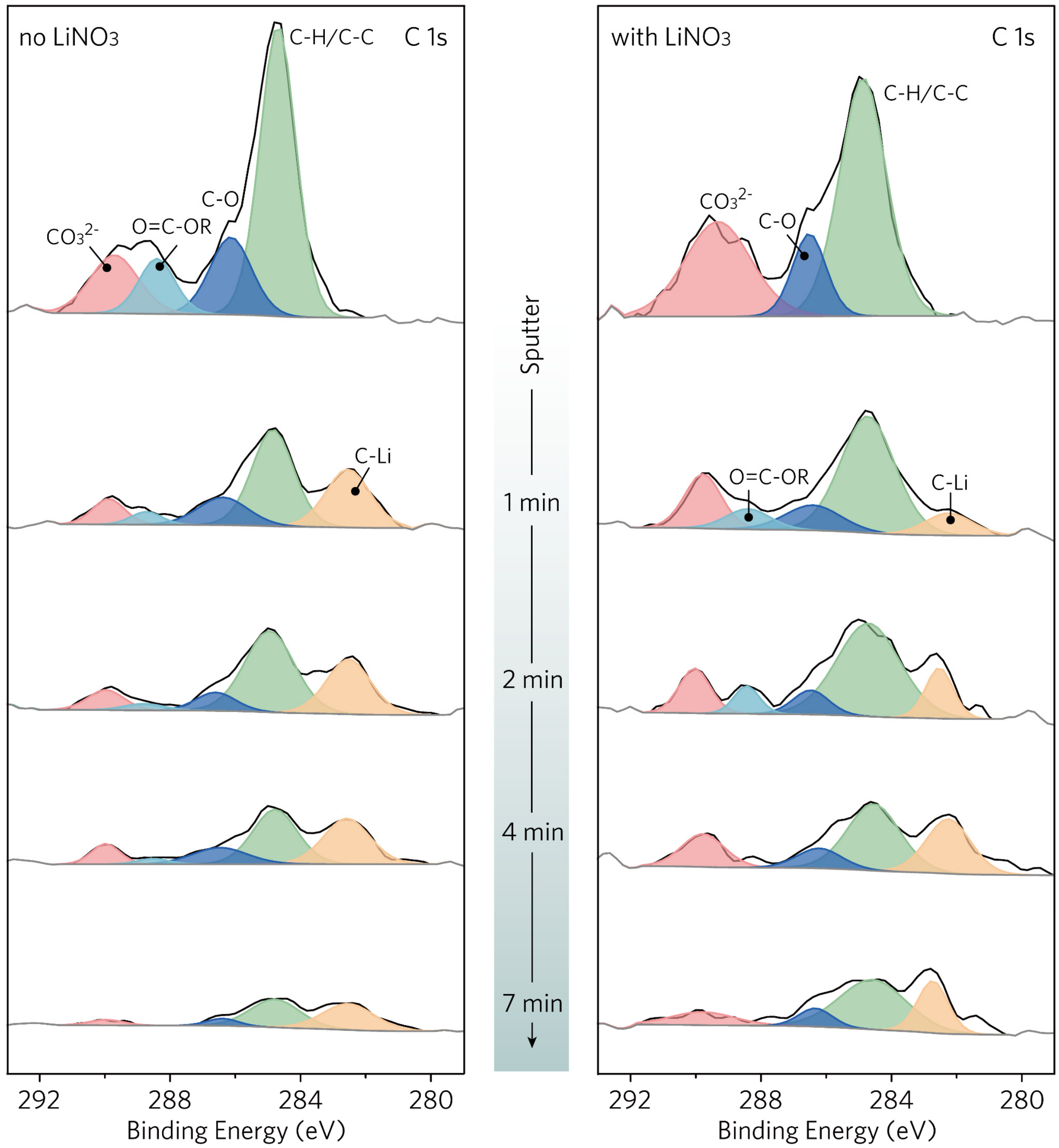

Supplementary Figure 25. XPS C_{1s} depth profiles of the SEI formed in 0.5 M $LiPF_6$ EC/DEC electrolyte with or without nitrate additive. Li deposition was carried out at a current density of 1 mA cm^{-2} and a capacity of 0.1 mAh cm^{-2} on Cu foil.

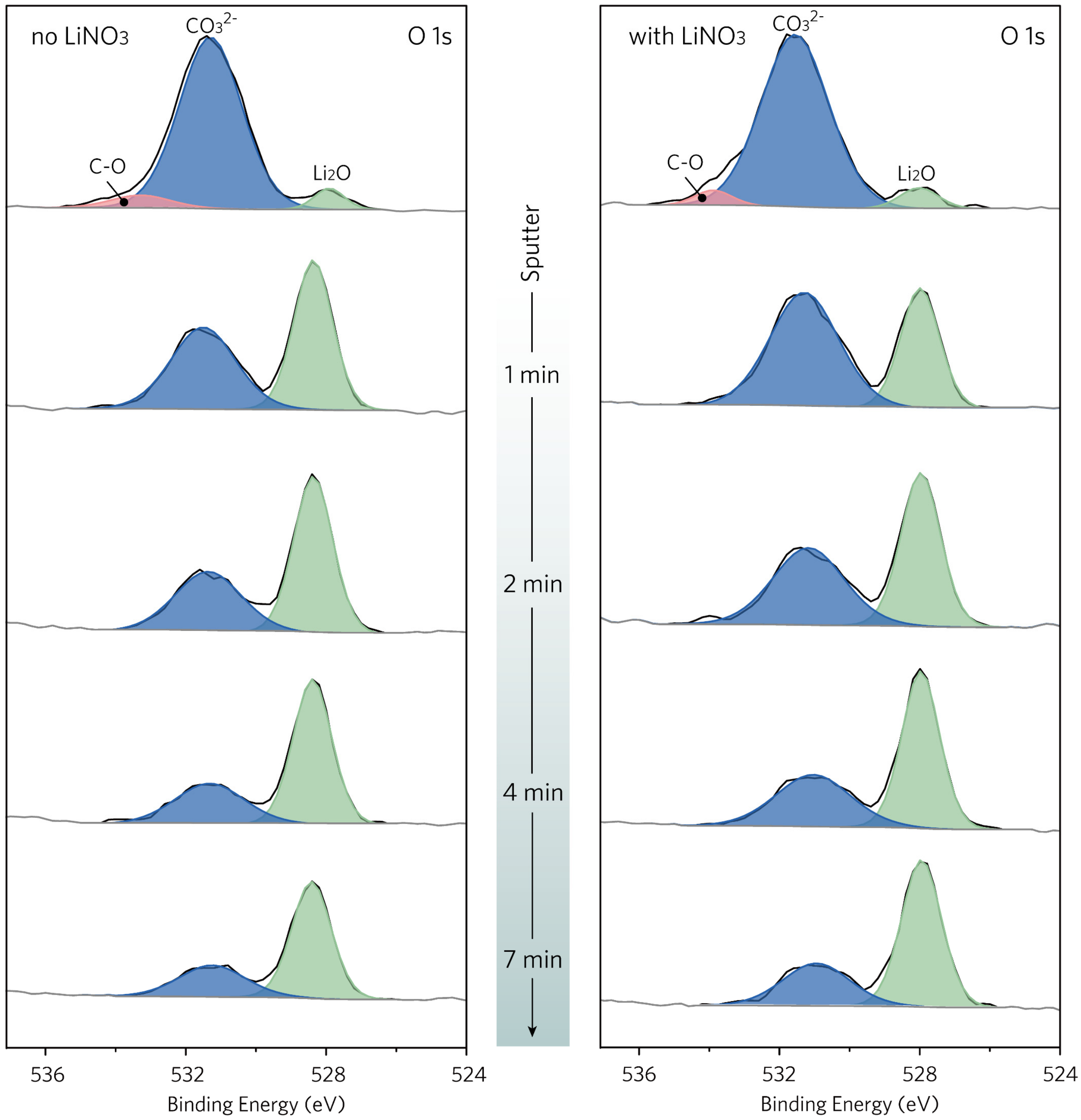

Supplementary Figure 26. XPS O_{1s} depth profiles of the SEI formed in 0.5 M $LiPF_6$ EC/DEC electrolyte with or without nitrate additive. Li deposition was carried out at a current density of 1 mA cm^{-2} and a capacity of 0.1 mAh cm^{-2} on Cu foil.

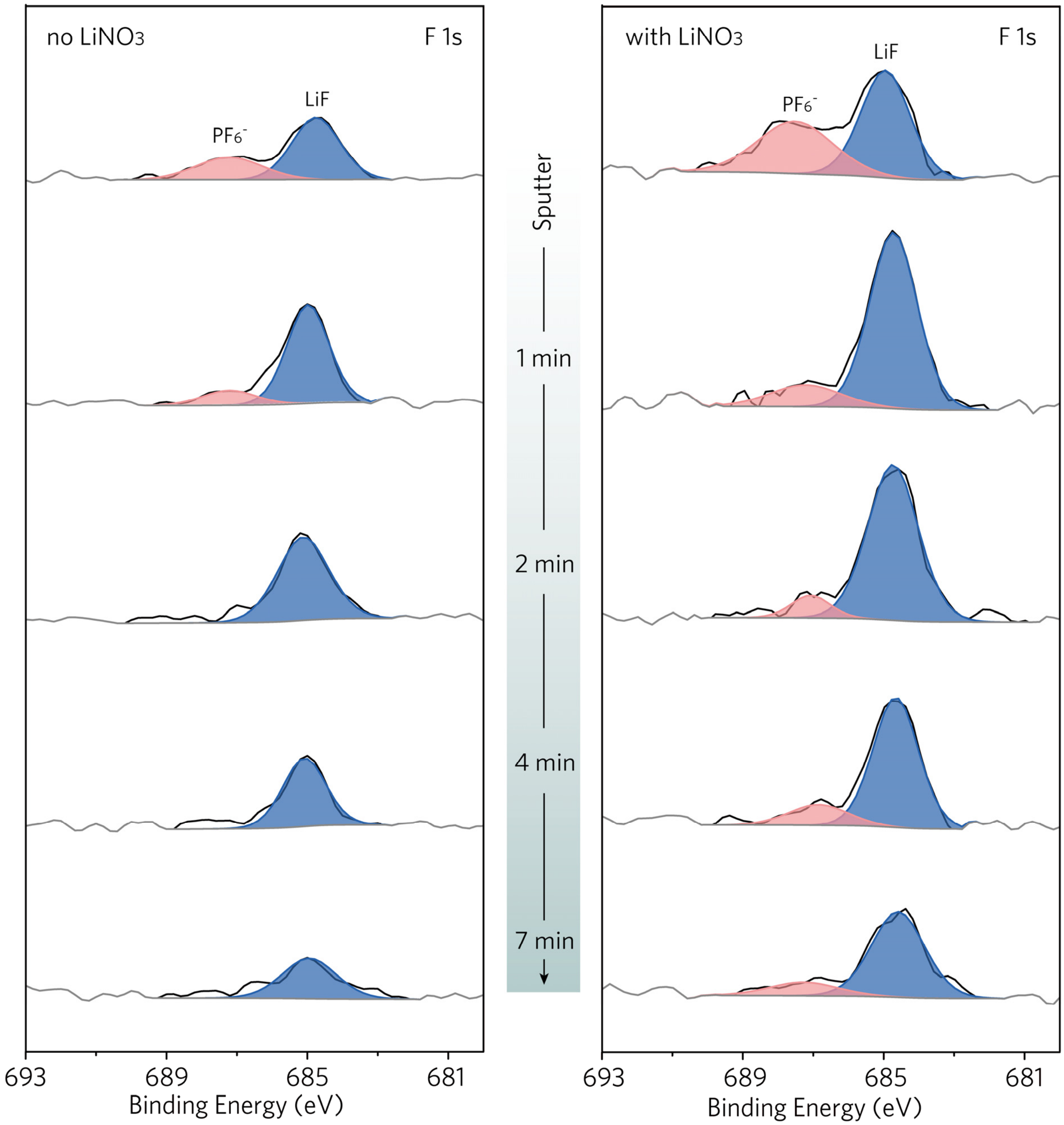

Supplementary Figure 27. XPS F_{1s} depth profiles of the SEI formed in 0.5 M $LiPF_6$ EC/DEC electrolyte with or without nitrate additive. Li deposition was carried out at a current density of 1 mA cm^{-2} and a capacity of 0.1 mAh cm^{-2} on Cu foil.

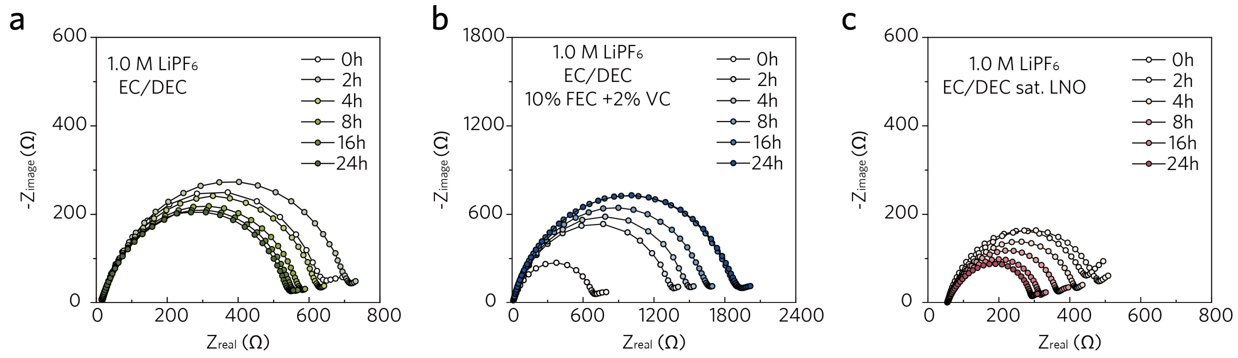

Supplementary Figure 28. EIS spectra of Li||Li symmetric cells as a function of storage time under open circuit potential condition in different electrolytes. (a) 1.0 M $LiPF_6$ EC/DEC electrolyte, and in 1.0 M $LiPF_6$ EC/DEC electrolyte with (b)10% FEC and 2% VC, or (c) $LiNO_3$ as additive.

Supplementary Methods

Supplementary Method 1: Calculating the mass loading of $LiNO_3$ in the LNO-SRF.

The average weight of ten pieces of LNO-SRF (~1.76 cm^2) was measured to be ~4.375 mg. The polymer to $LiNO_3$ weight ratio of LNO-SRF is 2 to 1. Therefore, the areal mass loading of $LiNO_3$ inside a coin cell is ~1.46 mg. If 80 μl electrolyte is added in each coin cell, the $LiNO_3$ additive mass loading is ~1.8 wt%, which is comparable to that in ether-based electrolytes.

Supplementary Method 2: Exchange current measurement using ultramicroelectrode.

Homebuilt microelectrodes using 25 μm tungsten (W) wire embedded in glass were used. Microelectrodes were polished with 100 nm grit lapping disks and cleaned after each test. Electrochemical cells consisted of a W microelectrode (working electrode) and Li foil (counter & reference electrode) submerged in electrolyte. CV scans were swept at 200 mV s^{-1} from 1 V to –270 mV (with $LiNO_3$) or –400 mV (without $LiNO_3$) and back to 0.5 V. Exchange current densities were calculated by fitting Tafel slopes of CV scans in the Li deposition direction. Raw data was filtered using a Savitsky-Golay filter to remove noise, and the capacitive current was corrected. The Tafel plot of the log current vs. cell polarization was linearly fit over the voltage range –150 mV to –200 mV to extract the Tafel slope. The exchange current was then calculated based on the intercept of the Tafel slope and the y-axis. Reported exchange current densities are calculated using 6 independent experiments using fresh microelectrode surfaces.
